# Island woodiness underpins accelerated disparification in plant radiations

**DOI:** 10.1111/nph.15797

**Published:** 2019-04-10

**Authors:** Nicolai M. Nürk, Guy W. Atchison, Colin E. Hughes

**Affiliations:** ^1^ Department of Plant Systematics Bayreuth Centre of Ecology and Environmental Research (BayCEER) University of Bayreuth Universitätsstrasse 30 95440 Bayreuth Germany; ^2^ Department of Systematic & Evolutionary Botany University of Zurich Zollikerstrasse 107 8008 Zurich Switzerland

**Keywords:** adaptive radiation, Andes, disparification, ecological opportunity, Hawaiian archipelago, Macaronesia (including Canary Islands), secondary (insular) woodiness, tropical alpine sky islands

## Abstract

- The evolution of secondary (insular) woodiness and the rapid disparification of plant growth forms associated with island radiations show intriguing parallels between oceanic islands and tropical alpine sky islands. However, the evolutionary significance of these phenomena remains poorly understood and the focus of debate.
- We explore the evolutionary dynamics of species diversification and trait disparification across evolutionary radiations in contrasting island systems compared with their nonisland relatives. We estimate rates of species diversification, growth form evolution and phenotypic space saturation for the classical oceanic island plant radiations – the Hawaiian silverswords and Macaronesian *Echium* – and the well‐studied sky island radiations of *Lupinus* and *Hypericum* in the Andes.
- We show that secondary woodiness is associated with dispersal to islands and with accelerated rates of species diversification, accelerated disparification of plant growth forms and occupancy of greater phenotypic trait space for island clades than their nonisland relatives, on both oceanic and sky islands.
- We conclude that secondary woodiness is a prerequisite that could act as a key innovation, manifest as the potential to occupy greater trait space, for plant radiations on island systems in general, further emphasizing the importance of combinations of clade‐specific traits and ecological opportunities in driving adaptive radiations.

## Abstract 2

See also the Commentary on this article by Baldwin, **224**: 8–10.

## Introduction

Evolutionary radiations – episodes of accelerated species diversification and/or trait disparification – are ubiquitous across organismal groups in diverse geographical and ecological settings (Davis *et al*., 2005; Linder, 2008; Glor, 2010; Hughes *et al*., 2015; Cardillo *et al*., 2017; Hutter *et al*., 2017). All of these settings potentially present ecological opportunities, the new adaptive zones of Simpson (1953), long thought to be the main factor driving radiations (Williams, 1969; Carlquist, 1974; Pincheira‐Donoso *et al*., 2015; Stroud & Losos, 2016). Ecological opportunities can cause diversifying selection whereby adaptation is driving ecomorphological diversification of species; a process known as adaptive radiation (Osborn, 1902; Simpson, 1944, 1953; Schluter, 2000).

The idea that ecological opportunity is the key driver of radiations is particularly compelling for oceanic island systems (Lack, 1947; Losos, 1994; Böhle *et al*., 1996; Baldwin & Sanderson, 1998; Givnish, 2000; Losos & Ricklefs, 2009; Grant & Grant, 2011). Island‐like systems (Itescu, 2018) such as lakes (Fryer, 1969; Seehausen, 2015), caves (Gillespie, 2004; Wessel *et al*., 2013) and mountains often present similar ecological opportunities, potentially driving radiations in these systems (Knowles, 2000; Kadereit & von Hagen, 2003; Hughes & Eastwood, 2006; Nürk *et al*., 2013b; Uribe‐Convers & Tank, 2015; Gehrke *et al*., 2016; Pouchon *et al*., 2018). Despite the centrality of extrinsic opportunity for radiations on island and island‐like systems, quantitative comparative analyses of ecological opportunities across different radiations have rarely been undertaken. In this study we attempt to gain new insights into the importance of ecological opportunity in radiations by revisiting the striking parallels between plant radiations on oceanic islands and tropical alpine montane ‘sky islands’ (Smith & Young, 1987; Warshall, 1995; Gehrke & Linder, 2014), parallels which have intrigued evolutionary biologists ever since Carlquist's (1965, 1974) classical studies of island biology.

The insular attributes of montane sky islands include isolation and dispersal limitation by a surrounding, often nonpermeable matrix (Itescu, 2018), limited area, pronounced ecological boundaries and strong elevational gradients, all of which contribute to high species endemism (Janzen, 1967; Simpson, 1974; Luteyn, 1999; Särkinen *et al*., 2012; Hughes & Atchison, 2015; Irl *et al*., 2017). Indeed, in the first two pages of Island Life, Carlquist (1965) drew attention to the island‐like attributes of mountains, referring to them as ‘islands of the upper air’. Tropical alpine sky islands and oceanic islands also share climatic moderation (Carlquist, 1965): the buffering effect of open oceans that results in extended year‐round growing seasons on islands is analogous to the equable aseasonal climates in tropical alpine sky islands, where year‐round growing seasons also prevail, albeit tempered by low mean annual temperatures with high diurnal fluctuations (Hedberg, 1964; Luteyn, 1999; Nürk *et al*., 2018). Climatic moderation has been suggested to prompt evolution of longer plant life cycles in island species compared with their mainland relatives, a long‐recognized phenomenon known as insular woodiness (Darwin, 1859; Wallace, 1878; Carlquist, 1974; Dulin & Kirchoff, 2010; Whittaker *et al*., 2017).

Although insular woodiness has sometimes been viewed as the likely ancestral state with woody continental sister lineages driven to extinction, we now know that insular woodiness has evolved secondarily in many plant lineages on oceanic islands (Böhle *et al*., 1996; Francisco‐Ortega *et al*., 1997; Baldwin *et al*., 1998; Baldwin, 2007; García‐Maroto *et al*., 2009; Carine *et al*., 2010; Lens *et al*., 2013). More recently, shifts from annual to perennial and/or herbaceous to secondarily woody life‐history strategies have also been associated with shifts from lowland to montane continental habitats (Tank & Olmstead, 2008; C. S. Drummond *et al*., 2012; Jabbour & Renner, 2012; Karl & Koch, 2013; Kostikova *et al*., 2013; Roquet *et al*., 2013; Gehrke *et al*., 2016; Kidner *et al*., 2016; Neupane *et al*., 2017). These findings suggest that secondary woodiness (a broader term including secondary insular woodiness) is more prevalent than previously realized, and that the majority of instances of secondary woodiness are not necessarily restricted to oceanic islands (Hughes & Atchison, 2015).

Secondary woodiness and montane perenniality have been implicated as possible key evolutionary innovations driving radiations in island systems. Carine *et al*. (2010) showed that the most diverse Macaronesian plant radiations are disproportionately those that developed secondary woodiness, suggesting that this might be triggering their radiation. This is in line with the low proportion of annual plant species, the high proportion of chamaephytes and the notable occurrences of arborescence in endemic island floras (Lems, 1961; Luteyn, 1999). Similarly, C. S. Drummond *et al*. (2012) proposed that the shift from annual to perennial life history provided a key adaptation (Hughes & Atchison, 2015; Nevado *et al*., 2016), triggering accelerated species diversification in montane western New World *Lupinus*. A similar link between perenniality, montane habitats and radiations was suggested for Delphinieae (Ranunculaceae) in the Himalayas (Jabbour & Renner, 2012), and *Lachemilla* and *Alchemilla* (Rosaceae) in the Andes and the African mountains (Gehrke *et al*., 2016; Morales‐Briones *et al*., 2018).

**Figure 1:**
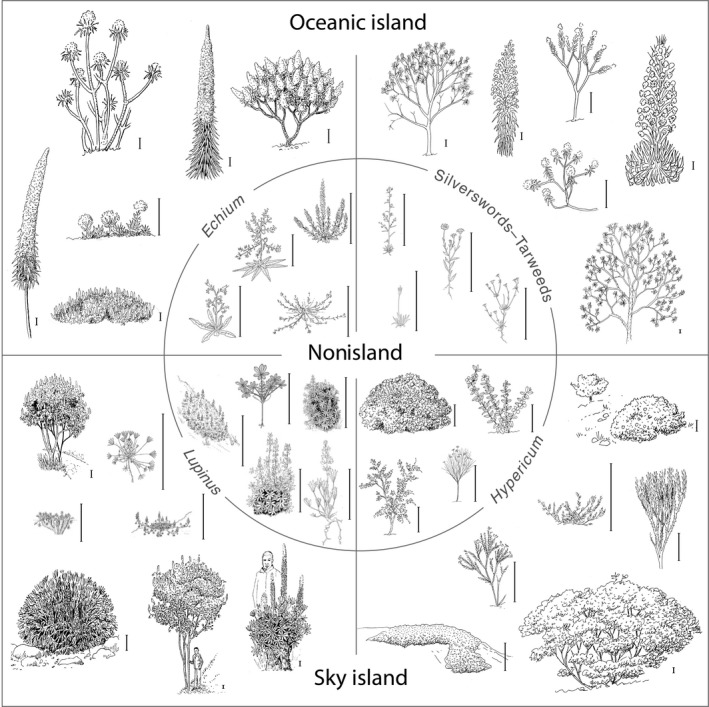
Disparity of growth forms and plant size variation among island and nonisland taxa in the four study groups – island *Echium* (clockwise from top left): *E*. *giganteum*,* E. wildpretii*,* E. leucophaeum*,* E. brevirame*,* E. hierrense*,* E. pininana*; nonisland *Echium* (clockwise from top right): *E. vulgare*,* E. creticum*,* E. horridum*,* E. asperrimum*; island silverswords (clockwise from top left): *Dubautia knudsenii*,* Argyroxiphium grayanum*,* Dubautia menziesii*,* Argyroxiphium sandwicense*,* Dubautia reticulata*,* Dubautia waialealae*; nonisland silverswords (clockwise from top left): *Calycadenia villosa*,* Layia jonesii*, *Madia stebbinsii*,* Raillardella argentea*; sky island *Hypericum* (clockwise from top right): *H. phellos*,* H. juniperinum*,* H. laricifolium*,* H. prostratum*,* H. mexicanum*,* H. brevistylum*; nonisland *Hypericum* (clockwise from top left): *H. kalmianum*,* H. crux‐andrae*,* H. gentianoides*,* H. galioides*; sky island *Lupinus* (clockwise from top left): *L. jelskianus*,* L. subacaulis*,* L. buchtienii*,* L. weberbaueri*,* L*. *semperflorens*,* L. cuzcensis*,* L. pulvinaris*; nonisland *Lupinus* (clockwise from top left): *L. angustifolius*,* L. microcarpus*,* L. cosentinii*,* L. luteus*,* L. albus*. [Correction added after online publication 10 April 2019: species names have been corrected.]

Much emphasis has been placed on secondary woodiness and montane perenniality in island systems (Dulin & Kirchoff, 2010; Lens *et al*., 2013; Garcia‐Verdugo *et al*., 2014), but little has been done to understand the rapid disparification (divergent phenotypic evolution) of growth forms associated with plant radiations in island systems (Hughes & Atchison, 2015). Growth form, and hence plant size, is a key functional trait and a leading component of plant ecological strategies (Stebbins, 1957), potentially triggering species diversification (Boucher *et al*., 2017) and/or disparification (Hughes & Atchison, 2015). Many oceanic and sky island plant radiations display highly diverse growth forms, including prostrate woody mat plants, cushion plants, arborescent shrubs, subshrubs, giant acaulescent and stem rosettes, single trunk trees and woody lianas (Fig. 1; Böhle *et al*., 1996: fig. 3; Baldwin, 1997, 2003: fig. 3.1; Hughes & Eastwood, 2006: fig. 2; Nürk *et al*., 2013b: fig. 1; Hughes & Atchison, 2015: fig. 1; Diazgranados & Barber, 2017: fig. 1). This disparity of growth forms has been amply described from oceanic and tropical alpine sky islands (Lems, 1961; Carlquist, 1965, 1974; Shmida & Werger, 1992; Böhle *et al*., 1996; Baldwin, 1997; Nürk *et al*., 2013b; Hughes & Atchison, 2015; Gehrke *et al*., 2016; Diazgranados & Barber, 2017; Pouchon *et al*., 2018), and probably results from selection‐driven adaptive evolution into different ecological niches during radiation (C. S. Drummond *et al*., 2012). However, rates of growth form evolution for island clades have not been quantified or compared with their nonisland relatives, even though wide sampling of mainland relatives is essential for estimating evolutionary rates, detecting rate shifts and understanding trajectories of diversification on islands (Haines *et al*., 2014). More generally, the association between accelerated disparification and intensified species diversification on island systems remains to be tested.

Here, we investigate the factors underpinning oceanic and sky island radiations, focusing on the impact of secondary woodiness and the disparification of plant growth forms. We analyse rates of species diversification and trait disparification for island clades compared with their nonisland relatives and compare oceanic and sky island systems. Specifically, we test two hypotheses:

1. Secondary woodiness is a key adaptation for island radiations facilitating disparification of plant growth forms. Under this hypothesis we expect that woody life histories are derived in the study clades (secondary woodiness) and that shifts from herbaceous to woody life histories are closely associated with shifts to higher rates of diversification and dispersal to island systems.
2. Island radiations are driven by ecological opportunity in the sense that lineages experience opportunities for adaptive divergence as a result of the availability of resources in the island environment. In this context, we expect island clades to show accelerated rates of disparification and larger trait spaces as compared with their nonisland relatives.

To test our hypotheses, we analysed classical oceanic island plant radiations (Hawaii and Macaronesia) and two well‐studied tropical Andean sky island radiations. Using time‐calibrated phylogenies comprehensively sampling both island and nonisland taxa, and qualitative and quantitative data on life history and growth form, we applied model selection and model‐averaging using Bayesian and maximum likelihood (ML) approaches to estimate rates and compare models of trait disparification and species diversification in island clades and their nonisland relatives.

## Materials and Methods

### Study groups, time trees and trait data

We selected two oceanic island radiations, Macaronesian *Echium* L. (Boraginaceae) and the Madiinae Benth. & Hook.f. (Asteraceae), which includes the Hawaiian silversword alliance and tarweeds from California and the California islands (Channel Islands, Guadalupe, and San Benito Islands), hereafter silverswords–tarweeds, plus two well‐studied Andean sky island radiations, *Hypericum* L. (Hypericaceae) and *Lupinus* L. (Fabaceae). For each group we used the best sampled time tree (C. S. Drummond *et al*., 2012; Nürk *et al*., 2018) or reconstructed a phylogeny using published sequence data and estimated divergence times under relevant age constraints using an uncorrelated lognormal relaxed‐clock model in beast v.1.8 (A. J. Drummond *et al*. (2012); for details about sampled taxa, DNA sequence loci, and fossil constraints see Supporting Information Table S1; Methods S1).

Data on species distributions, that is occurrence on island (oceanic/sky island) vs nonisland (continental/lowland), and trait data on life history (herbaceous/annual vs woody/perennial) and growth forms (plant size) were assembled from the literature (Bramwell, 1972; Robson, 1987, 1990; Carlquist *et al*., 2003; Baldwin *et al*., 2012; C. S. Drummond *et al*., 2012; Talavera *et al*., 2012), herbarium specimen data, and field observations (by the authors and B. G. Baldwin, pers. comm., 2018). Because the definition of sky island boundaries is not necessarily straightforward, we relied on clade‐specific definitions based on biogeography, including elevation (see ‘Island radiations are underpinned by ecological opportunity’ in the Discussion section). To infer evolutionary trajectories of life‐history strategy and secondary woodiness, it must be borne in mind that the associations and boundaries between annual vs perennial, iteroparous vs semelparous, herbaceous vs woody and generation times are often fuzzy and sometimes decoupled. For example, many perennial plants are herbaceous, some woody perennials flower and fruit in the first year with short generation times, some long‐lived plants are monocarpic, boundaries between herbaceous and woody are not always clear‐cut (Dulin & Kirchoff, 2010), and secondarily woody ‘perennials’ are often relatively short‐lived and sometimes semelparous (Lens *et al*., 2013). Using these categories as a proxy for life history needs to be done carefully. For *Lupinus*, the shift from annual to perennial life history is well documented (C. S. Drummond *et al*., 2012), but some montane perennials are predominantly herbaceous plants, especially in the Rockies. For this reason, we scored species of *Lupinus* as annual or perennial. For *Hypericum*, the majority of New World species are woody shrubs (Nürk *et al*., 2013a), but several (annual or perennial) herbaceous species occur in North and South America. In *Echium* and the silverswords–tarweeds, herbaceous/woody growth forms correspond closely with annual/perennial life histories, with very few exceptions (e.g. *Echium vulgare*,* Kyhosia bolanderi*). We thus scored species of *Echium*,* Hypericum* and silverswords–tarweeds as either herbaceous or woody.

To assess growth form disparity and estimate disparification rates we relied on plant size, specifically mean height. Plant size alone does not fully capture the disparity within the study clades because different growth forms can have similar plant heights (Fig. 1), and hence size alone probably underestimates interspecific variation. It does, however, provide a suitable proxy (Ackerly, 2009; Harmon *et al*., 2010; Giometto *et al*., 2013), which can be used across disparate clades (Boucher *et al*., 2017). All data used are deposited in Dryad (https://doi.org/10.5061/dryad.rt530k9).

### Estimating rates of species diversification

To identify radiations, we tested for shifts in diversification rates by combining Bayesian model averaging (BMA) with exploratory Bayesian approaches. First, we fitted various pure‐birth and birth‐death models over trees with *a priori* assignments of island/nonisland clades or tree partitions. Models were specified to test for rate differences between island and nonisland clades by linking/unlinking speciation and extinction rate parameters among specified clades (Table S2). Incomplete taxon sampling was accounted for by specifying clade sampling fractions based on taxonomic revisions (Bramwell, 1972; Carlquist *et al*., 2003; Baldwin *et al*., 2012; C. S. Drummond *et al*., 2012; Robson, 2012; Talavera *et al*., 2012). For each model we ran a Markov chain Monte Carlo (MCMC) for one million generations, with write frequencies every 1000 generations, and estimated the log marginal likelihood using default parameters in bayesrate v.1.65 (Silvestro *et al*., 2011). We used BMA to generate a joint posterior distribution of parameter estimates by resampling the models’ individual posterior samples based on their relative probability and specifying 10% as burn‐in. We compared model fit with the data using Bayes factors (BFs) following Kass & Raftery (1995).

To further explore diversification rate heterogeneity across time trees and identify shifts at phylogenetic positions not considered in BMA, we used the Bayesian mixture model approach in bamm 2.5 (Rabosky, 2014a), while being aware of the debate on statistical concerns (Meyer *et al*., 2018; Rabosky, 2018). Sampling fractions were specified at the lowest taxonomic level possible (Table S3). Rate priors were set to the scale of the trees using ‘setBAMMpriors’ in BAMMtools v.2.1.6 (Rabosky, 2014b) in R v3.4.3 (R Core Team, 2013) with the regime shift prior set to 1.0, placing strong prior probability on zero shifts. We ran four Metropolis coupled MCMC chains for 20 million generations, with write frequencies every 10 000 generations, specifying 30% as burn‐in. Parameter convergence was assessed based on likelihood traces and effective sample size (≫ 200) using Coda v.0.19.1 (Plummer *et al*., 2006) in R. The posterior distribution of rate regime shifts was compared with the prior (results not shown), and the 95% credible set of shift configurations identified using BAMMtools.

### Estimating the origins of secondary woodiness

We assessed evolutionary trajectories of life‐history strategy across the study clades using stochastic character mapping (SCM; Huelsenbeck *et al*., 2003) and ML ancestral state estimation, focusing on the phylogenetic positions of shifts from herbaceous/annual to woody/perennial life histories within each group. We generated 1000 stochastic character maps (simmap trees) for the binary character life history (herbaceous/annual vs woody/perennial) using MCMC to sample character histories from their posterior probability distributions. Before, we tested goodness of fit to the data of different transition rate probability matrices using the likelihood ratio test: a one‐parameter Mk model with equal transition rates among the character states (ER) and an all‐rates‐different Mk model. Simmap trees were summarized to display posterior densities of each state across branches and nodes for the mapped character, life history. Ancestral states obtained by ML and posterior probabilities from SCM were compared to check congruence. Analyses used the R functions *ace* in ape v.5.0 (Paradis *et al*., 2004) and ‘make.simmap’ in phytools v.0.6‐44 (Revell, 2011).

### Estimating mode and rate of disparification (phenotypic evolution)

To approximate the influence of ecological opportunity on island radiations, we estimated rates of disparification of growth forms (mean plant height), explicitly testing whether growth form has evolved differently in island and nonisland clades. We combined model selection and model averaging approaches in a ML framework to test the mode of trait evolution in island vs nonisland clades and to assess goodness‐of‐fit weighted estimates of rates of phenotypic evolution (in continuous trait models the stochastic variance term or rate parameter *σ* ^2^; Butler & King, 2004; Ackerly, 2009; Kaliontzopoulou & Adams, 2016). Our analyses fit single and multiregime unconstrained Brownian motion (BM) and constrained Ornstein–Uhlenbeck (OU) continuous trait models of evolution on log_e_‐transformed mean plant heights using *a priori* assigned island and nonisland ‘regimes’, where regimes are clades or tree partitions that potentially underwent different trait evolution trajectories (Cressler *et al*., 2015). For silverswords–tarweeds, we assigned species from Hawaii and the California islands as two separate regimes. For assignment we used stochastic character mapping, accounting for uncertainty by generating 100 simmap trees for each study group for the binary state character island/nonisland using the approach described earlier (see ‘Estimating the origins of secondary woodiness’ in the Materials and Methods section).

We fitted the following six models each over 100 simmap trees per study group: an unconstrained single rate model (BM1) and a constrained single optimum model (OU1), assuming no difference between island and nonisland regimes (null hypotheses); a BM model with variable phenotypic evolutionary rates and a single root value (BM*σ* ^2^) assuming separate rates between the regimes; a model with different trait means per regime (the state/optimum parameter *θ*; OU*θ*), assuming different plant height optima per regime; a model with different state means and variably strong constraints towards regime optima (the ‘pull to the optima’ parameter *α*; OUθ*α*), assuming differently directed evolution towards different plant height optima in island and nonisland regimes; and a model with multiple rate and optima parameter values (OUθ*σ* ^2^) assuming different evolutionary rates causing phenotypic differences in the regimes. To assess the individual model performance in each run, we relied on eigen decomposition of the Hessian matrix, in which negative eigenvalues indicate poor parameter estimation, and so excluded these. We also excluded ‘failed’ models, in which log‐likelihood values of two to three order of magnitudes greater were estimated; these estimates are potentially a result of model failure, whereby complexity of the model can be greater than the information contained in the data, resulting in one or more poorly estimated parameters and a spurious log‐likelihood (Cressler *et al*., 2015). To evaluate confidence in the estimated parameter values, we conducted parametric bootstraps on the six models to calculate 95% confidence intervals (95% CI). Analyses used the ‘OUwie’ and ‘OUwie.boot’ function in R's OUwie v.1.5 package (Beaulieu & O'Meara, 2015).

We compared goodness of fit with the data for the six models using sample size‐corrected Akaike information criterion (AICc), also calculating AICc differences (∆AICc) and AICc weights (*w*) based on relative likelihoods (Burnham & Anderson, 2002). We assumed that the best model tested had the lowest AICc score (highest *w* value). The AICc weight for a given model is the relative likelihood of the model divided by the sum of all relative likelihoods across all models and was used to obtain model‐averaged estimates of parameters (Posada & Buckley, 2004). We calculated *w* for each model in each run and averaged parameter and bootstrap estimates over all models and runs. To obtain a distribution of parameter estimates, we calculated *w* for each model per run and averaged parameter estimates over the six models per run using R (R Core Team, 2013).

### Estimating trait space saturation

To further investigate the influence of ecological opportunity, we tested the hypothesis that island species evolved greater growth form trait space compared with their nonisland relatives. We compared rates of trait (mean plant height) space saturation between island and nonisland clades or tree partitions. To do this, we used ‘trait saturation’ analysis (TSA) to compare evolutionary trajectories of disparity between clades standardized for clade‐specific limits of evolutionary change (Rolshausen *et al*., 2018). The method uses phenotypic (continuous trait) and phylogenetic (cophenetic) pairwise distances between all taxa in the tree to identify relative disparity maxima at a certain divergence time delineating the trait space saturation curve (‘saturation function’) which illustrates the trajectory of trait evolution in a linearly standardized 0–1 space (pairwise distance relative to maximum distance in the tree; where 0 = no disparity/no divergence, and 1 = maximal disparity/maximal divergence time). A linearly increasing saturation curve indicates neutral trait evolution following BM, whereas exponential curves indicate early burst patterns, and logarithmic curves the evolution of disparity among recently diverged species. Because we are comparing evolutionary growth form trajectories between (nested) island clades and (remaining) nonisland species, we used a ‘nested clade’ version of TSA, calculating the saturation function per clade (island or nonisland) relative to that of the entire tree, that is we linearly adjusted individual saturation functions relative to the trait space of the entire tree. We evaluated confidence around saturation functions by means of 1000 rounds of jackknife resampling, each time randomly pruning 10% of the taxa. Overlap between the island/nonisland saturation functions was quantified along the *x*‐axis and tested for congruence using a two‐sided Kolmogorov–Smirnov (KS) test. TSA analyses were based on the R functions from Rolshausen *et al*. (2018).

## Results

### Rates of species diversification

**Figure 2:**
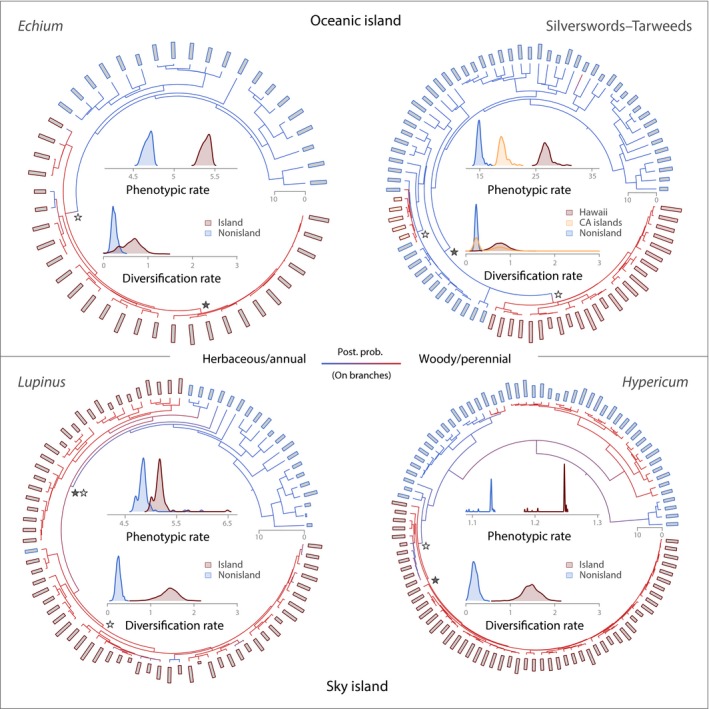
Origin of secondary woodiness, phenotypic rates of plant height evolution and species diversification comparing oceanic and sky island clades (denoted by red bar outlines) with their nonisland relatives (denoted by blue bar outlines). Bars at tips of trees denote mean plant height in log_e_(cm). Coloured branches of phylogenetic trees indicate the evolution of life history (blue, herbaceous/annual; red, woody/perennial) inferred using stochastic character mapping. Asterisks denote diversification rate shifts (inferred using bamm; filled, most credible shift configuration; unfilled, second most credible configuration). Time axes for phylogenetic trees are in Myr. The central insets show density plots of model‐averaged rates of plant height evolution (above) and net diversification (below), comparing island clades (red; California island (CA island) species are in orange) with nonisland relatives (blue).

Diversification rates are higher in oceanic and sky island clades than in their continental or lowland relatives (Fig. 2). In sky island clades, averaged net species diversification rates increase by more than an order of magnitude (e.g. in *Hypericum*, net diversification rates are: nonisland = 0.19 (95% highest posterior density (HPD): 0.05–0.34); sky island = 1.47 (1.09–1.83)), whereas in oceanic island clades, median rates are two to three times higher than in their mainland relatives (Table 1). All four study groups except the silverswords–tarweeds show significant support for lineage diversification being different (unlinked) between the island and nonisland taxa (Table S2). For *Echium* there is positive support (BF = 4.6) for a model in which diversification rates are unlinked between the Macaronesian clade and the mainland taxa, compared with the best model which assumes island and nonisland rates to be the same (linked). For the silverswords–tarweeds., there is no significant evidence for different diversification rates, although six unlinked models fit the data better than the best linked model (BF < 1.5). For the Andean sky island radiations, *Hypericum* and *Lupinus*, all tested models assuming different diversification rates between lowland and sky island clades obtained higher log marginal likelihoods than any linked model, with a BF for the best‐scoring unlinked model against the best linked model indicating very strong support (*Hypericum*, BF = 24.2; *Lupinus*, BF = 54.2). This difference is mirrored in the combined posterior sample obtained by BMA, where density plots of net diversification rates between sky island and nonisland taxa do not overlap, whereas in the oceanic island radiations they do overlap, most notably for the tarweeds clade from the California islands (Fig. 2).

**Table 1:** Comparison of island and nonisland clade ages, species richness, mean growth height, and rates of species diversification and phenotypic evolution

Clade	Crown age (95% HPD) (Myr)	Species richness (sampled)	Net diversification (95% HPD)	Growth height (range) (m)	Phenotypic optimum *θ* (95% CI)	Adaptation/pull rate *α* (95% CI)	Phenotypic rate *σ* ^2^ (95% CI)
*Echium*							
Nonisland	11.5 (9.9–17.8)	28 (71%)	0.24 (0.13–0.37)	0.58 (0.21–0.95)	0.59 (0.50–0.69)	9.19 (2.22–27.91)	4.68 (1.06–15.51)
Oceanic island	4.2 (3.6–8.0)	28 (100%)	0.65 (0.26–1.00)	1.14 (0.25–2.50)	1.09 (0.97–1.32)	9.16 (2.21–27.80)	5.37 (1.20–17.66)
*Hypericum*							
Nonisland	20.7 (17.0–24.7)	85 (58%)	0.19 (0.05–0.34)	0.54 (0.10–2.40)	0.52 (0.43–0.60)	1.10 (0.51–1.53)	1.13 (0.65–1.64)
Sky island	3.1 (2.0–4.3)	99 (57%)	1.47 (1.09–1.83)	0.70 (0.12–3.14)	0.61 (0.51–0.83)	1.10 (0.51–1.53)	1.24 (0.74–1.81)
*Lupinus*							
Nonisland	9.2 (4.1–12.4)	27 (78%)	0.25 (0.14–0.35)	0.21 (0.02–0.65)	0.21 (0.16–0.29)	2.52 (1.38–24.10)	4.86 (2.49–45.31)
Sky island	4.6 (1.7–5.6)	190 (33%)	1.44 (0.99–1.78)	0.53 (0.02–5.50)	0.55 (0.45–0.67)	2.52 (1.38–24.06)	5.18 (2.73–47.70)
Silverswords–tarweeds (Madiinae)							
Nonisland	14.7 (14.0–15.9)	78 (73%)	0.23 (0.18–0.30)	0.39 (0.08–1.28)	0.39 (0.35–0.44)	17.49 (4.52–23.98)	12.92 (3.17–19.09)
Oceanic island (CA Islands)	1.1 (1.0–3.1)	5 (100%)	0.49 (0.16–1.00)	0.32 (0.10–0.75)	0.33 (0.21–1.67)	17.49 (4.52–23.98)	18.68 (2.27–36.74)
Oceanic island (Hawaii)	3.6 (2.7–6.2)	29 (86%)	0.77 (0.42–1.13)	2.17 (0.25–8.00)	2.22 (1.67–2.93)	17.49 (4.52–23.98)	29.45 (6.84–45.67)

CA Islands, California islands. Net diversification rates (speciation – extinction Myr^−1^) obtained by Bayesian model averaging detailing the median and the 95% highest posterior density (HPD) interval (in brackets; bayesrate); Ouwie model parameters (*σ* ^2^, *α*,* θ*) obtained by Akaike weighted model averaging with the 95% confidence interval (95% CI) obtained by a parametric bootstrap (in brackets). Note that the phenotypic optimum estimates correspond to mean plant height (in m).

Exploratory analyses of rate heterogeneity among the lineages also indicate that diversification regime shifts in the silverswords–tarweeds are not exclusively related to occurrence on islands. Although a rate shift is estimated by the Bayesian mixture model approach at the crown node of the silverswords radiation on Hawaii, two additional shifts at nodes not related to dispersal to islands are detected in the silverswords–tarweeds (Fig. 2). In *Echium*,* Hypericum* and *Lupinus*, estimated diversification regime shifts coincide with dispersal to oceanic or sky islands, with additional diversification regime shifts nested within the island clades (Fig. 2).

### Origin of secondary woodiness

Evolutionary trajectories of life‐history strategy identify shifts from herbaceous/annual to woody/perennial life histories at derived positions, clearly indicating concordant evolution of secondary woodiness in all four study groups (Fig. 2). The ER model for ancestral state estimation is always selected as fitting best, and SCM and ML estimates are congruent (Figs S1–S4). The ancestral condition is estimated to be herbaceous/annual in all groups, although with ambiguity in *Hypericum* (posterior probabilities at the root: herbaceous, 0.57; woody, 0.43). Changes from herbaceous/annual to secondarily woody/perennial life histories are, on average, less frequent than changes in the opposite direction in *Echium* (1.27 (95% credible interval: 1.02–3.10) vs 2.19 (1.20–4.10) events) and especially *Lupinus* (2.97 (0.57–6.27) vs 8.96 (5.88–13.57)). In *Hypericum*, changes are almost balanced (herbaceous to woody, 5.43 (2.27–8.25); woody to herbaceous, 5.01 (2.22–9.42)). In the silverswords–tarweeds, reversal from the secondarily woody state to herbaceous is almost absent (herbaceous to woody, 3.06 (3.01–4.08); woody to herbaceous, 0.10 (0.01–1.50)). In the tarweeds from the California islands, the origin of secondary woodiness is estimated to have arisen one node before the crown node of the California island taxa (Figs 2, S4). In the remaining island clades across all four study groups, the evolution of secondary woodiness coincides with diversification rate shifts (Fig. 2).

### Mode and rate of disparification (phenotypic evolution)

Analyses of growth form evolution find clear support in all four study groups for constrained OU models over unconstrained BM models (Table S4). We did not, however, find support for a particular OU model within and among groups. For *Echium* and *Lupinus*, the best model tested is an OU*θ* model with different trait optima for the nonisland and island regimes (*Echium*, ∆AICc = 1.6; *Lupinus,* ∆AICc = 1.9). For *Hypericum* the best fit is an OU1 model (∆AICc = 0.6). An OUθ*σ* ^2^ model is the best fit for the silverswords–tarweeds (∆AICc = 1.9; Table S4).

Phenotypic evolutionary rate estimates averaged using AICc weights indicate disparification of growth forms to be higher in island clades than in their continental or lowland relatives (Fig. 2). Although rate differences are generally pronounced, for example, in the Hawaiian silversword radiation (*σ* ^2^ = 29.5) compared with its mainland relatives (*σ* ^2^ = 12.9), these are not significant, with the 95% CIs obtained by bootstrapping overlapping among island and nonisland species in all four groups (Table 1). In *Hypericum*, rate estimates are low, with little gap between rate densities (Fig. 2) mainly because of low variance among runs (Table 1). The phenotypic optimum values indicate significantly taller plant height optima for island clades except for *Hypericum* and the California island tarweeds (Table 1).

### Trait space saturation

**Figure 3:**
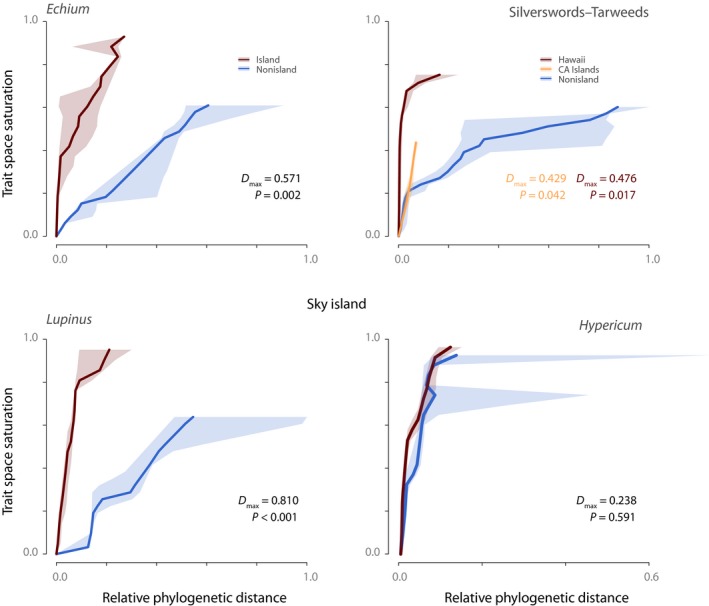
Trajectories of growth form evolution assessed though trait saturation analysis. Trait space saturation and relative phylogenetic distance represent the space spanned for the entire phylogeny, with a proportional projection of the saturation curves for the island (dark red line) and nonisland (blue line) pruned phylogenies. Shaded areas around saturation curves indicate the 95% confidence interval inferred by means of jackknife resampling. The value of the test statistic (*D*_max_) and the *P*‐value of the test are indicated. CA islands, California islands. Note that 1.0 indicates maximum distance, in trait disparity (relative pairwise distance) and in divergence times (relative cophenetic distance).

Trajectories of growth form evolution indicate evolution of larger trait spaces in mean plant height in island clades compared to their nonisland relatives in *Echium*,* Hypericum*,* Lupinus* and Hawaiian silverswords, but not in the tarweeds from the California islands (Fig. 3). Notably, all island clades show rapid filling of trait space, that is, trait space saturation is achieved within small relative phylogenetic distances (i.e. within short divergence times, such that growth form disparity is high among closely related species), indicating significantly accelerated rates of disparification in all island clades (KS test, *P *<* *0.05; all except *Hypericum*; Fig. 3).

## Discussion

Lineage‐specific extrinsic ecological opportunities associated with newly emerged oceanic islands, the new adaptive zones of Simpson (1953), have long been considered to be the key driver of species, trait and ecological diversification that characterize island radiations (Losos, 2010). Similar opportunities have also been central to explaining rapid diversification in continental island‐like formations such as lakes (Seehausen, 2015) and mountains (Hughes & Atchison, 2015), where some of the most spectacular evolutionary radiations occurred (Madriñán *et al*., 2013). In this study, we estimate rates of species diversification and trait disparification in the comparative context of their mainland relatives for the emblematic oceanic island plant radiations, Macaronesian *Echium* and Hawaiian silverswords, for the first time (Landis *et al*., 2018). We also present evidence of striking parallels between oceanic and tropical alpine sky island systems, demonstrating accelerated evolutionary rates across plant radiations in both systems following the evolution of ‘island woodiness’.

### Are secondary woodiness and perenniality key innovations for island radiations?

Across flowering plants, evolutionary shifts from woody to herbaceous (or perennial to annual) life history strategies have occurred frequently (Tank & Olmstead, 2008) and have been associated with shifts to higher rates of molecular evolution (Smith & Donoghue, 2008; Yang *et al*., 2015), accelerated species diversification (Boucher *et al*., 2017), and accelerated niche evolution (Smith & Beaulieu, 2009; Ogburn & Edwards, 2015). These increased rates of evolution have been attributed to shorter generation times in annual/herbaceous/smaller‐sized species (Boucher *et al*., 2017). On islands and island‐like systems, however, many aspects of plant evolution run counter to these general patterns. In all four study groups, we found evidence that species diversification rates, growth form disparity and rates of disparification are higher in the secondarily woody/perennial island clades than in their herbaceous/annual nonisland relatives (Fig. 2; Table 1).

We found clear evidence that secondary woodiness is closely associated with dispersal to islands, either oceanic or sky island, in all four study clades. Thus, we cannot reject our first hypothesis that secondary woodiness is a key adaptation for island radiations, acknowledging that we included only four well‐selected plant clades. The fact that island clades mostly occupy greater trait space than their nonisland relatives (Fig. 3) is probably a result of the different adaptive potentials of annuals vs perennials or herbaceous vs woody species (King, 1990; Koch *et al*., 2004). In terms of plant size variation, perennial species are inherently more evolvable, with potential to occupy greater trait space than annuals, which are biophysically more constrained. This greater growth form disparity of island woody perennials compared with nonisland herbaceous annuals (Fig. 3) also opens up greater potential trait space in other plant life‐history characteristics, such as the number and sizes of inflorescences, as evidenced by the large compound inflorescences with numerous flowers in some Hawaiian silverswords (e.g. *Argyroxiphium sandwicense* ssp. *macrocephalum*, with inflorescences up to 2 m high and with up to 600 capitula, each with as many as 600 disc florets; Fig. 1) (Baldwin, 2003), Macaronesian *Echium* and Andean *Lupinus*, where a > 100‐fold disparity in numbers of flowers per inflorescence is evident (e.g. *Lupinus weberbaueri*, with inflorescences comprising > 500 flowers vs *L. pulvinaris*, with less than five flowers; C. E. Hughes, unpublished). [Correction added after online publication 10 April 2019: species names have been corrected in the preceding sentence.] Longer life cycles and the potential for more flowers on island plants compared with their herbaceous mainland colonists were the traits that prompted Wallace's (1878) longevity hypothesis to explain insular woodiness. Disparification of growth forms thus has many ramifications for evolutionary diversification of island clades.

That secondary woodiness opens up greater potential trait spaces is in line with life‐history theory, which predicts that shorter annual life cycles will be favoured in seasonal environments (Roff, 2002; Franks *et al*., 2007). Conversely, perennial life‐history strategies are advantageous in aseasonal, more mesic, cooler montane environments, where lower temperatures, lower relative growth rates and high rates of seed mortality select against annuals (Ogburn & Edwards, 2015). These predictions are borne out by the many plant clades showing close associations between life history and elevation, with annuals in lowlands and perennials at higher elevations (Evans *et al*., 2009; Smith & Beaulieu, 2009; Ogburn & Edwards, 2015). The examples of secondary woodiness outside oceanic islands conform to this pattern of montane perenniality (C. S. Drummond *et al*., 2012; Jabbour & Renner, 2012; Kidner *et al*., 2016; Neupane *et al*., 2017), suggesting that island woodiness/montane perenniality is a general evolutionary pattern for plants (Ogburn & Edwards, 2015). Secondary island woodiness releases constraints on body size and life cycle suffered by annual or herbaceous species simply as a result of their shorter overall growth periods. This may enable woody/perennial plant lineages to evolve novel phenotypes that interact with their environments in new ways via evolution of diverse growth forms (Ackerly, 2009; Evans *et al*., 2009; Fig. 1). In this sense, secondary woodiness could be viewed as a key evolutionary innovation that drives disparification in island radiations. Although this is a compelling hypothesis, the effects of secondary woodiness are difficult to separate from wider ecological opportunities on islands, or from the possible effects of differences in population sizes between island and nonisland species (Schrieber & Lachmuth, 2017). Nevertheless, it is secondary woodiness that allows species to explore greater trait space and potential niches and, hence, to contribute to diversification in island and island‐like systems.

### Secondary woodiness is driven mainly by moderate climates on island systems

Explanations for insular woodiness have been debated since Darwin (1859), Wallace (1878) and Carlquist (1974) put forward their competition, longevity and climatic moderation hypotheses. Our results show that radiations on oceanic islands and tropical alpine sky islands follow parallel trajectories of accelerated trait and species diversification associated with secondary perenniality/secondary woodiness, as suggested by Carlquist (1965, 1974). The repeatability of these patterns of growth form disparification across the superficially very different contexts of oceanic and sky islands, which show remarkably similar evolutionary patterns, suggests a common underlying cause and offers the potential to shed new light on the competing hypotheses of Darwin (1859), Wallace (1878) and Carlquist (1974). Carlquist (1965, 1974) suggested that insular woodiness is favoured in aseasonal, frost‐free island climates that permit year‐round plant growth. Lack of marked temperature seasonality in low‐ and mid‐elevation habitats in Macaronesia and Hawaii compared with the more seasonal mainland habitats where relatives of *Echium* and island silverswords and tarweeds grow, fits with Carlquist's hypothesis. Tropical alpine zones of the Andes, the East African mountains (Gehrke & Linder, 2009) and New Guinea (Goetsch *et al*., 2011) also share aseasonal and relatively uniform climates throughout the year, whereas the nonisland relatives of our sky island study clades occur in highly seasonal environments in North America. As far as we are aware, documented examples of secondary woodiness, often associated with accelerated disparification of growth forms, are generally confined to environments that offer year‐round growing (temperature) conditions, such as those found in (sub)tropical oceanic islands or tropical alpine mountains (Carlquist, 1974; Lens *et al*., 2013; Hughes & Atchison, 2015; Kidner *et al*., 2016; Neupane *et al*., 2017). This remarkable coincidence between oceanic islands and tropical sky islands, alongside the apparent lack of secondary woodiness and associated plant growth form disparification in island radiations in seasonal environments, provide compelling support for Carlquist's climatic moderation hypotheses to explain secondary woodiness and growth form disparification in these contrasting island systems.

### Island radiations are underpinned by ecological opportunity

As studies of island radiations are extended, it seems ever clearer that many facets of island evolution buck the general trends in plant life‐history evolution. The common rules of more rapid diversification of species and growth forms associated with annuals are reversed on islands. There is also limited evidence to suggest that rates of molecular evolution in island perennials are higher than closely related herbaceous and generally more slowly evolving nonisland sister groups, in both tropical sky island (Nevado *et al*., 2016) and oceanic island clades (Barrier *et al*., 2001). It is noteworthy that these results contrast with the generally lower rates of molecular evolution in woody lineages compared with their herbaceous relatives (Smith & Donoghue, 2008). We suggest that these ‘devious pathways of island evolution’ (Carlquist, 1965) are the evolutionary manifestations of the common ground underpinning island radiations, namely ecological opportunity.

Almost all sampled oceanic and sky island species are secondarily woody/perennial and their nonisland close relatives herbaceous or annual, but there are a few interesting exceptions. First, in *Hypericum*, although rates of species diversification and growth form evolution are higher in the sky island clade, overall disparity of growth forms is only marginally greater (Fig. 3). This is a result of reliance on mean heights as a proxy for growth form (Notes S1) and the similar disparity of growth forms in a woody North America clade (Nürk *et al*., 2018). Second, as indicated earlier (see the Materials and Methods section), *Lupinus* species were coded as annual or perennial (rather than herbaceous or woody), because many North American species are herbaceous perennials. This lineage diversified rapidly across the Rocky Mountain sky island in line with ecological opportunity and driven by ecological factors such as high seedling mortality and low growth rates in cold temperatures or poorly developed soils. Furthermore, the greatest growth form disparity in *Lupinus* has evolved in the aseasonal tropical alpine Andean sky island, in line with Carlquist's hypothesis of climatic moderation. Third, there are a few reversals to herbaceous life history within the Andean sky island clade of *Hypericum* (e.g. *H. pratense*,* H. silenoides*) and to annual species within the Andean clade of *Lupinus* (e.g. *L. huigrensis*,* L. lindleyanus*; Figs S2, S3). These ‘reversed’ (tertiary herbaceous/annual) species occur at lower elevations than the perennial woody species, within seasonally drier inter‐Andean valleys and on the progressively drier, mid‐elevation, Pacific flanks of the Andes, with one species, the ephemeral annual *L. mollendoensis*, occurring in dry Lomas formations close to the coast of Peru. These reversals to annual/herbaceous life history associated with shifts back to lowland habitats are perhaps analogous to back‐colonizations to the mainland nested within oceanic island radiations (Carine *et al*., 2004).

Finally, the woody tarweed *Deinandra minthornii*, a localized mainland endemic, is sister to the California island tarweeds, such that the transition from herbaceous to woody apparently preceded dispersal to the California islands (Figs 2, S4). Additionally, *D. martirensis*, a perennial with a woody base (Baldwin, 2003), was designated as nonisland (although nested within the Californian island clade; Fig. S4) because it occurs in mainland Baja California. The relationships of this species remain ambiguous (Baldwin, 2007; B. G. Baldwin, pers. comm.) but, despite being a mainland species, perenniality in *D. martirensis* is still associated with occurrence on an ‘island’, in this case the sky island of the Sierra San Pedro Martir (Baldwin, 2007). Our phenotypic evolutionary rates analyses suggest that the California island tarweeds show somewhat accelerated rates of growth form disparification but little increase in species diversification rate and no evidence of greater disparity of growth forms compared with mainland tarweeds, perhaps because, for Guadalupe Island *Deinandras*, plant size is a poor surrogate for growth form disparification (B. G. Baldwin, pers. comm.). Also, our analyses suggest limited diversification in the Californian island clade in line with an incipient radiation suggested by Carlquist (1965) and Baldwin (2007), and with the small island area and limited environmental heterogeneity (Warren *et al*., 2015) on Guadalupe Island. Taken together, these exceptions emphasize that it is combinations of intrinsic clade‐specific trait innovation and extrinsic opportunities that dictate diversification.

In this study, we included four exemplar clades, but many other island plant radiations also show high growth form disparity and high rates of diversification associated with secondary woodiness, including *Sonchus* in Macaronesia (Kim *et al*., 1999, 2007), *Dendroseris* in the Juan Fernández islands (Kim *et al*., 2007), Hawaiian lobelioids (Givnish, 2000; Givnish *et al*., 2009), other Andean sky island radiations, including Espeletiineae (Diazgranados & Barber, 2017; Pouchon *et al*., 2018), *Valeriana* (Bell & Donoghue, 2005; Moore & Donoghue, 2007; Bell *et al*., 2012; see Eriksen, 1989) and *Draba* (Karl & Koch, 2013), and tropical alpine plant radiations elsewhere (Gehrke & Linder, 2014; Schwery *et al*., 2015). However, despite the abundance of clades showing these patterns, detailed evolutionary rates analyses for a larger sample of island clades are needed to assess the generality of these findings and to test the hypothesis that a radiating clade on an oceanic or sky island is underpinned by both accelerated rates of phenotypic evolution and species diversification.

## Author contributions

CEH, GWA and NMN designed the research; GWA and NMN performed the research; CEH and NMN discussed the results; and all authors contributed to writing the manuscript.

## Supporting information

Please note: Wiley Blackwell are not responsible for the content or functionality of any Supporting Information supplied by the authors. Any queries (other than missing material) should be directed to the *New Phytologist* Central Office.

**Fig. S1** Time tree for *Echium* with species and clades, life‐history mappings.
**Fig. S2** Time tree for *Hypericum* with species and clades, life‐history mappings.
**Fig. S3** Time tree for *Lupinus* with species and clades, life‐history mappings.
**Fig. S4** Time tree for silverswords–tarweeds with species and clades, life‐history mappings.
**Methods S1** Clade‐specific information on age estimation and trait data.
**Notes S1** Details on potential biases using mean plant height in *Hypericum*.
**Table S1** Species data (distribution, life history, plant height) and DNA sequence GenBank numbers.
**Table S2** Diversification rate analysis: model specification and model fit.
**Table S3** Sampling fractions used in exploratory rate heterogeneity analysis.
**Table S4** Growth form: phenotypic (plant height) evolution analysis detailing model fit.
