# Supplementary material for: Island woodiness underpins accelerated disparification in plant radiations
Source: New Phytol. 2019 Apr 10;224(1):518–31. doi: 10.1111/nph.15797 (PMC6766886; doi:10.1111/nph.15797)
Supplement: Supplementary file 1 — Fig. S1 Time tree for Echium with species and clades, life‐history mappings. Fig. S2 Time tree for Hypericum with species and clades, life‐history mappings. Fig. S3 Time tree for Lupinus with species and clades, life‐history mappings. Fig. S4 Time tree for silverswords–tarweeds with species and clades, life‐history mappings. Methods S1 Clade‐specific information on age estimation and trait data. Notes S1 Details on potential biases using mean plant height in Hypericum. Table S1 Species data (distribution, life history, plant height) and DNA sequence GenBank numbers. Table S2 Diversification rate analysis: model specification and model fit. Table S3 Sampling fractions used in exploratory rate heterogeneity analysis. Table S4 Growth form: phenotypic (plant height) evolution analysis detailing model fit. [file NPH-224-518-s001.pdf]

***New Phytologist* Supporting Information**

# Island woodiness underpins accelerated disparification in plant radiations

Nicolai M. Nürk, Guy W. Atchison, Colin E. Hughes

Manuscript accepted: 12 March 2019

The following Supporting Information is available for this article:

**Table S1 Voucher** – *Information on species and sequence references included in the study, and coding of characters (distribution, life history, and growth form).*

**Table S2 Comparative diversification rate analysis**: *model specification and model fit (BayesRate).*

**Table S3 Sampling fractions**: *exploratory rate heterogeneity analysis (BAMM).*

**Table S4 Plant height**: *growth form (phenotypic) evolution analysis detailing model fit (OUwie).*

**Figures S1–S4 Secondary woodiness** – *time trees incl. tip names and clade assignment detailing life history analysis comparing ML to SCM ancestral state estimations (ace & make.simmap), and island/non-island mappings for* Echium *(Fig. S1),* Hypericum *(Fig. S2),* Lupinus *(Fig. S3), and Silverswords–Tarweeds (Fig. S4).*

**Methods S1 Clade specific tree and trait data** – *Phylogenetic reconstruction and age estimation (BEAST), fossils used for calibration, full references for trait data.*

**Notes S1 *Hypericum* traits** – *details on potential biases using mean plant height in the group.*

**Table S1**. Voucher – Information on taxa included in the study detailing species names per study group, clade assignment, mean plant height, coding of distribution (i, island; n, non-island) and life history traits (h, herbaceous; w, woody), and ENA/NCBI accession numbers of molecular marker used for phylogenies newly reconstructed in this study.

| | | Plant | Distri- | Life | Molecular marker | | | | |
|---|---|---|---|---|---|---|---|---|---|
| **Clade** | **Taxon** | **height** | **bution** | **history** | **ITS** | **D6DES** | **rbcL** | **trnL** | **trnLF** |
| ***Echium*** | | | | | | | | | |
| E | *E. acanthocarpum* Svent. | 170.0 | i | w | EU048853 | DQ315582 | — | EU433604 | EU433619 |
| E | *E. aculeatum* Poiret | 130.0 | i | w | EU048849 | DQ315568 | EU599868 | EU433600 | EU433615 |
| D | *E. albicans* Lag. & Rodr. | 56.0 | n | h | L43172 | — | — | L43170 | L43171 |
| C | *E. angustifolium* Mill. | 83.0 | n | h | — | — | EU599871 | EU600047 | EU599959 |
| A | *E. arenarium* Guss. | 37.5 | n | h | EU919584 | — | — | — | — |
| D | *E. asperrimum* Lam. | 65.0 | n | h | L43176 | — | — | L33347 | L43175 |
| E | *E. auberianum* Webb & Berthel. | 80.0 | i | w | L43180 | DQ315596 | EU599866 | EU600042 | EU599954 |
| E | *E. bethencourtii* Santos | 250.0 | i | w | — | DQ315595 | — | — | — |
| E | *E. bonnetii* Coynci | 25.0 | i | h | L43184 | DQ315578 | EU599864 | EU600040 | EU599952 |
| E | *E. brevirame* Spr. & Hutch. | 60.0 | i | w | L43188 | DQ315594 | — | L43186 | L43187 |
| E | *E. callithyrsum* Webb ex Bolle | 72.0 | i | w | L43196 | DQ315573 | — | L43194 | L43195 |
| E | *E. candicans* L.f. | 162.5 | i | w | EU048856 | DQ315587 | — | EU433607 | EU433622 |
| D | *E. creticum* L. | 57.5 | n | h | FJ763249 | — | — | L43206 | L43207 |
| E | *E. decaisnei* Webb & Berthel. | 135.0 | i | w | EU048852 | DQ315593 | EU599867 | EU600043 | EU433618 |
| E | *E. gentianoides* Webb ex Coynci | 70.0 | i | w | — | DQ315575 | — | — | — |
| E | *E. giganteum* L.f. | 250.0 | i | w | L43224 | DQ315567 | EU599870 | EU600046 | EU599958 |
| E | *E. handiense* Svent. | 102.5 | i | w | L43220 | DQ315577 | — | L43218 | L43219 |
| E | *E. hierrense* Webb ex Bolle | 160.0 | i | w | EU048848 | DQ315581 | — | EU433599 | EU433614 |
| D | *E. horridum* Batt. | 83.0 | n | h | L43228 | — | — | L43226 | L43227 |
| A | *E. humile* Desf. | 21.0 | n | h | AF284109 | — | — | AF284105 | AF284099 |
| E | *E. hypertropicum* Webb | 100.0 | i | w | EU048858 | DQ315592 | — | EU433609 | EU433624 |
| D | *E. italicum* L. | 59.5 | n | h | L43236 | — | EU599874 | EU600051 | EU599963 |
| E | *E. leucophaeum* Webb ex Spr. & Hutch. | 150.0 | i | w | L43240 | DQ315588 | EU599865 | EU600041 | EU599953 |
| D | *E. lusitanicum* L. | 87.0 | n | h | EU048847 | — | — | EU433598 | EU433613 |
| E | *E. nervosum* Dryand. | 130.0 | i | w | EU048855 | DQ315574 | — | EU433606 | EU433621 |
| E | *E. onosmifolium* Webb & Berthel. | 120.0 | i | w | L43260 | DQ315572 | — | L43258 | L43259 |
| A | *E. parviflorum* Moench | 25.0 | n | h | L43264 | — | — | L43262 | L43263 |
| E | *E. pininana* Webb & Berthel. | 78.5 | i | w | L43268 | DQ315586 | — | L43266 | L43267 |
| E | *E. pitardii* A. Chev | 25.0 | i | h | L43322 | DQ315569 | — | L43320 | L43321 |
| D | *E. plantagineum* L. | 66.0 | n | h | L43272 | — | EU599872 | EU600049 | EU599960 |
| D | *E. pycnanthum* Pomel | 21.0 | n | h | AF284108 | — | — | AF284104 | AF284101 |
| D | *E. rosulatum* Lange | 78.0 | n | h | L43276 | — | — | L43274 | L43275 |
| B | *E. russicum* J.F. Gmel. | 82.5 | n | h | L43280 | — | — | L33356 | L43279 |

*continued*

| Clade | Taxon | Plant height | Distri-bution | Life history | Molecular marker | | | | |
|---|---|---|---|---|---|---|---|---|---|
| | | | | | ITS | D6DES | rbcL | trnL | trnLF |
| A | *E. sabulicola* Pomel | 37.5 | n | h | L43288 | DQ315570 | — | L43286 | L43287 |
| E | *E. simplex* DC | 215.0 | i | w | EU048851 | DQ315584 | HM849965 | EU433602 | EU433617 |
| E | *E. stenosiphon* Webb | 100.0 | i | w | EU048859 | DQ315591 | — | EU433611 | EU433627 |
| E | *E. strictum* L.f. | 130.0 | i | w | L43292 | DQ315590 | — | L43290 | L43291 |
| E | *E. sventenii* Bramw. | 145.0 | i | w | | DQ315579 | — | — | — |
| A | *E. tenue* Lam. | 83.0 | n | h | AF284106 | DQ315571 | — | AF284102 | AF284098 |
| E | *E. triste* Svent. | 60.0 | i | h | L43324 | DQ315576 | — | — | L43323 |
| D | *E. tuberculatum* Hoffmanns. & Link | 95.0 | n | h | L43300 | — | — | L43298 | L43299 |
| A | *E. vilmorinianum* Sauvage & Vindt | 83.0 | n | h | AF284107 | — | — | AF284103 | AF284100 |
| E | *E. virescens* Bolle | 165.0 | i | w | EU048850 | DQ315583 | EU599869 | EU433601 | EU433616 |
| E | *E. vulcanorum* A. Chev | 150.0 | i | w | EU048857 | DQ315589 | — | EU433608 | EU433623 |
| D | *E. vulgare* L. | 75.0 | n | h | AY092900 | — | KF158087 | L33362 | FJ789880 |
| E | *E. webbii* Coyncii | 170.0 | i | w | EU048854 | DQ315580 | — | EU433605 | EU433620 |
| E | *E. wildpretii* H.Pearson ex Hook.f. | 250.0 | i | w | KF287973 | DQ315585 | EU599863 | EU600039 | EU599951 |
| B | *Pontechium maculatum* (L.) Böhle & Hilger | 82.5 | n | h | EU919608 | — | — | KF288055 | — |
| ***Hypericum*** | | | | | | | | | |
| I | *H. aciculare* Kunth | 115.0 | i | w | | | | | |
| H | *H. acostanum* N.Robson | 45.0 | i | w | | | | | |
| I | *H. adpressum* W.P.C.Barton | 60.0 | n | h | | | | | |
| I | *H. andinum* Gleason | 30.0 | i | w | | | | | |
| B | *H. apocynifolium* Small | 55.0 | n | w | | | | | |
| H | *H. arbuscula* Stanley & Steyerm. | 35.0 | i | w | | | | | |
| D | *H. boreale* (Britton) Bickn. | 37.5 | n | h | | | | | |
| B | *H. brachyphyllum* (Spach) Steud. | 75.0 | n | w | | | | | |
| C | *H. brasiliense* Choisy | 65.0 | n | h | | | | | |
| H | *H. brevistylum* Choisy | 11.5 | i | h | | | | | |
| I | *H. bryoides* Gleason | 12.0 | i | w | | | | | |
| B | *H. buckleyi* M.A.Curtis | 25.0 | n | w | | | | | |
| I | *H. callacallanum* N.Robson | 35.0 | i | w | | | | | |
| C | *H. campestre* Cham. & Schltdl. | 125.0 | n | h | | | | | |
| D | *H. canadense* L. | 31.5 | n | h | | | | | |
| I | *H. cardonae* Cuatrec. | 60.0 | i | w | | | | | |
| I | *H. carinosum* R.Keller | 155.0 | i | w | | | | | |
| B | *H. chapmanii* W.P.Adams | 190.0 | n | w | | | | | |
| B | *H. cistifolium* Lam. | 90.0 | n | w | | | | | |
| C | *H. connatum* Lam. | 61.5 | n | h | | | | | |
| I | *H. costaricense* N.Robson | 55.0 | i | w | | | | | |

*continued*

| Clade | Taxon | Plant height | Distri-bution | Life history | Molecular marker | | | | |
|---|---|---|---|---|---|---|---|---|---|
| | | | | | ITS | D6DES | rbcL | trnL | trnLF |
| B | *H. crux_andreae* (L.) Crantz | 55.0 | n | w | | | | | |
| I | *H. cuatrecasii* Gleason | 160.0 | i | w | | | | | |
| I | *H. cymobrathys* N.Robson | 75.0 | i | w | | | | | |
| I | *H. decandrum* Turcz. | 35.0 | i | w | | | | | |
| B | *H. densiflorum* Pursh | 120.0 | n | w | | | | | |
| F | *H. dichotomum* Lam. | 13.0 | n | h | | | | | |
| B | *H. dolabriforme* Vent. | 32.5 | n | w | | | | | |
| F | *H. drummondii* (Grev. & Hook.) Torr. & A.Gray | 45.0 | n | h | | | | | |
| B | *H. fasciculatum* Lam. | 150.0 | n | w | | | | | |
| B | *H. frondosum* Michx. | 153.0 | n | w | | | | | |
| B | *H. galioides* Lam. | 100.0 | n | w | | | | | |
| I | *H. garciae* Pierce | 75.0 | i | w | | | | | |
| E | *H. gentianoides* (L.) Britton, Sterns & Poggenb. | 33.5 | n | h | | | | | |
| I | *H. gladiatum* N.Robson | 42.5 | i | w | | | | | |
| I | *H. gleasonii* N.Robson | 82.5 | i | w | | | | | |
| D | *H. globuliferum* R.Keller | 19.5 | n | h | | | | | |
| H | *H. gnidioides* Seem. | 79.5 | i | w | | | | | |
| I | *H. goyanesii* Cuatrec. | 200.0 | i | w | | | | | |
| D | *H. gramineum* G.Forst. | 61.0 | n | h | | | | | |
| I | *H. harlingii* N.Robson | 255.0 | i | w | | | | | |
| I | *H. hartwegii* Benth. | 60.0 | i | w | | | | | |
| I | *H. horizontale* N.Robson | 50.0 | i | w | | | | | |
| I | *H. humboldtianum* Steud. | 42.5 | i | w | | | | | |
| B | *H. hypericoides* (L.) Crantz | 65.0 | n | w | | | | | |
| I | *H. irazuense* Kunze ex N.Robson | 170.0 | i | w | | | | | |
| D | *H. japonicum* Thunb. | 26.0 | n | h | | | | | |
| I | *H. juniperinum* Kunth | 135.0 | i | w | | | | | |
| B | *H. kalmianum* L. | 40.0 | n | w | | | | | |
| D | *H. lalandii* Choisy | 29.5 | n | h | | | | | |
| I | *H. lancifolium* Gleason | 85.0 | i | w | | | | | |
| I | *H. lancioides* subsp. *congestiflorum* Cuatrec. | 55.0 | i | w | | | | | |
| I | *H. laricifolium* Juss. | 315.0 | i | w | | | | | |
| C | *H. linoides* A.St.-Hil. | 65.0 | n | h | | | | | |
| B | *H. lissophloeus* W.P.Adams | 240.0 | n | w | | | | | |
| I | *H. llanganaticum* N.Robson | 120.0 | i | w | | | | | |

*continued*

| Clade | Taxon | Plant height | Distri-bution | Life history | Molecular marker | | | | |
|---|---|---|---|---|---|---|---|---|---|
| | | | | | ITS | D6DES | rbcL | trnL | trnLF |
| B | *H. lloydii* (Svenson) W.P.Adams | 30.0 | n | w | | | | | |
| B | *H. lobocarpum* Gatt. | 120.0 | n | w | | | | | |
| H | *H. loxense* subsp. *loxense* Benth. | 60.0 | i | w | | | | | |
| I | *H. lycopodioides* Triana & Planch. | 200.0 | i | w | | | | | |
| I | *H. magniflorum* Cuatrec. | 175.0 | i | w | | | | | |
| I | *H. maguirei* N.Robson | 65.0 | i | w | | | | | |
| D | *H. majus* (A.Gray) Britton | 32.5 | n | h | | | | | |
| I | *H. marahuacanum* subsp. *marahuacanum* N.Robson | 90.0 | i | w | | | | | |
| I | *H. mexicanum* L. | 82.5 | i | w | | | | | |
| B | *H. microsepalum* (Torr. & A.Gray) A.Gray ex S.Watson | 42.5 | n | w | | | | | |
| D | *H. mutilum* L. | 37.5 | n | h | | | | | |
| I | *H. myricariifolium* Hieron. | 200.0 | i | w | | | | | |
| B | *H. myrtifolium* Lam. | 65.0 | n | w | | | | | |
| B | *H. nitidum* subsp. *exile* Lam. | 170.0 | n | w | | | | | |
| B | *H. nudiflorum* Michx. | 125.0 | n | w | | | | | |
| I | *H. parallelum* N.Robson | 35.0 | i | w | | | | | |
| I | *H. phellos* subsp. *phellos* Gleason | 215.0 | i | w | | | | | |
| H | *H. philonotis* Schltdl. & Cham. | 33.0 | i | w | | | | | |
| I | *H. pimeleoides* Planch. & Linden ex Triana & Planch. | 95.0 | i | w | | | | | |
| C | *H. polyanthemum* Klotzsch ex Reichardt | 25.0 | n | w | | | | | |
| H | *H. pratense* Schltdl. & Cham. | 45.0 | i | h | | | | | |
| B | *H. prolificum* L. | 112.5 | n | w | | | | | |
| I | *H. prostratum* Cuatrec. | 20.0 | i | w | | | | | |
| H | *H. quitense* R.Keller | 65.0 | i | w | | | | | |
| C | *H. rigidum* subsp. *rigidum* A.St.-Hil. | 125.0 | n | w | | | | | |
| I | *H. ruscoides* Cuatrec. | 80.0 | i | w | | | | | |
| I | *H. sabiniforme* Trevir. | 40.0 | i | w | | | | | |
| D | *H. scioanum* Chiov. | 20.0 | n | h | | | | | |
| I | *H. selaginella* N.Robson | 15.0 | i | w | | | | | |
| H | *H. silenoides* Juss. | 35.0 | i | h | | | | | |
| B | *H. sphaerocarpum* Michx. | 40.0 | n | w | | | | | |

*continued*

| Clade | Taxon | Plant height | Distri-bution | Life history | Molecular marker ITS | D6DES | rbcL | trnL | trnLF |
|---|---|---|---|---|---|---|---|---|---|
| I | *H. sprucei* N.Robson | 110.0 | i | w | | | | | |
| I | *H. strictum* Kunth | 60.0 | i | w | | | | | |
| I | *H. struthiolifolium* Juss. | 50.0 | i | w | | | | | |
| B | *H. suffruticosum* W.P.Adams | 10.0 | n | w | | | | | |
| B | *H. tenuifolium* Pursh | 30.0 | n | w | | | | | |
| C | *H. ternum* A.St.-Hil. | 45.0 | n | w | | | | | |
| G | *H. terrae-firmae* Sprague & Riley | 150.0 | i | w | | | | | |
| B | *H. tetrapetalum* Lam. | 60.0 | n | w | | | | | |
| I | *H. tetrastichum* Cuatrec. | 52.5 | i | w | | | | | |
| H | *H. thesiifolium* Kunth | 36.0 | i | h | | | | | |
| I | *H. thuyoides* Kunth | 200.0 | i | w | | | | | |
| I | *H. valleanum* N.Robson | 100.0 | i | w | | | | | |
| I | *H. woodianum* N.Robson | 150.0 | i | w | | | | | |
| A | *H. fraseri* (Spach) Gleason | 45.0 | n | h | | | | | |
| A | *H. fauriei* (Blume) Makino | 50.0 | n | h | | | | | |
| A | *H. virginicum* Raf. | 45.0 | n | h | | | | | |
| A | *H. walteri* (J.F.Gmel.) Gleason | 60.0 | n | h | | | | | |
| ***Lupinus*** | | | | | | | | | |
| P | *L. albicaulis* Douglas | 75.0 | i | w | | | | | |
| P | *L. albifrons* Benth. | 110.0 | i | w | | | | | |
| P | *L. andersonii* S.Watson | 57.0 | i | w | | | | | |
| P | *L. arboreus* Sims | 160.0 | i | w | | | | | |
| Q | *L. arcticus* S.Watson | 30.0 | i | w | | | | | |
| P | *L. argenteus* Pursh | 42.0 | i | w | | | | | |
| K | *L. arizonicus* (S.Watson) S.Watson | 30.0 | n | h | | | | | |
| S | *L. arvensis* Benth. | 40.0 | i | h | | | | | |
| R | *L. aschenbornii* S.Schauer | 40.0 | i | w | | | | | |
| S | *L. ballianus* C.P.Sm. | 160.0 | i | w | | | | | |
| S | *L. bangii* Rusby | 65.0 | i | h | | | | | |
| K | *L. benthamii* A.Heller | 45.0 | n | h | | | | | |
| O | *L. bicolor* Lindl. | 25.0 | n | h | | | | | |
| S | *L. bogotensis* Benth. | 170.0 | i | w | | | | | |
| I | *L. brevicaulis* S.Watson | 5.0 | n | h | | | | | |
| P | *L. breweri* A.Gray | 10.0 | i | w | | | | | |
| P | *L. cervinus* Kellogg | 20.0 | i | w | | | | | |
| S | *L. chachas* C.P.Sm. | 65.0 | i | w | | | | | |
| P | *L. chamissonis* Eschsch. | 125.0 | i | w | | | | | |
| S | *L. chrysanthus* Ulbr. | 60.0 | i | w | | | | | |
| J | *L. citrinus* Kellogg | 35.0 | n | h | | | | | |
| K | *L. concinnus* J.Agardh | 6.5 | n | h | | | | | |
| P | *L. duranii* Eastw. | 8.5 | i | w | | | | | |

*continued*

| Clade | Taxon | Plant height | Distri-bution | Life history | Molecular marker ITS | D6DES | rbcL | trnL | trnLF |
|---|---|---|---|---|---|---|---|---|---|
| R | *L. elegans* Kunth | 200.0 | i | w | | | | | |
| S | *L. ellsworthianus* C.P.Sm. | 80.0 | i | w | | | | | |
| P | *L. excubitus* M.E.Jones | 110.0 | i | w | | | | | |
| I | *L. flavoculatus* A.Heller | 6.0 | n | h | | | | | |
| P | *L. formosus* Greene | 50.0 | i | w | | | | | |
| P | *L. grayi* S.Watson | 27.0 | i | w | | | | | |
| N | *L. guadalupensis* C.P.Sm. | 40.0 | n | h | | | | | |
| J | *L. hirsutissimus* Benth. | 60.0 | n | h | | | | | |
| R | *L. huachucanus* M.E.Jones | 65.0 | i | w | | | | | |
| S | *L. huaronensis* J.F.Macbr. | 80.0 | i | w | | | | | |
| S | *L. huigrensis* Rose ex C.P.Sm. | 35.0 | i | h | | | | | |
| R | *L. jaimehintonianus* B.L. Turner | 400.0 | i | w | | | | | |
| P | *L. latifolius* J.Agardh | 67.0 | i | w | | | | | |
| P | *L. lepidus* Lindl. | 30.0 | i | w | | | | | |
| P | *L. leucophyllus* Lindl. | 57.0 | i | w | | | | | |
| S | *L. lindleyanus* J.Agardh | 18.0 | i | h | | | | | |
| P | *L. littoralis* Lindl. | 20.0 | i | w | | | | | |
| I | *L. luteolus* Kellogg | 65.0 | n | h | | | | | |
| S | *L. mantaroensis* C.P.Sm. | 31.0 | i | h | | | | | |
| R | *L. mexicanus* Lag. | 55.0 | i | w | | | | | |
| I | *L. microcarpus* Sims | 18.0 | n | h | | | | | |
| S | *L. microphyllus* Desr. | 4.0 | i | w | | | | | |
| S | *L. misticola* Ulbr. | 25.0 | i | w | | | | | |
| S | *L. mollendoensis* Ulbr. | 15.0 | i | h | | | | | |
| R | *L. montanus* Kunth | 80.0 | i | w | | | | | |
| S | *L. mutabilis* Sweet | 150.0 | i | h | | | | | |
| O | *L. nanus* Benth. | 25.0 | n | h | | | | | |
| P | *L. neomexicanus* Greene | 70.0 | i | w | | | | | |
| P | *L. nevadensis* A.Heller | 27.0 | i | w | | | | | |
| Q | *L. nootkatensis* Sims | 60.0 | i | w | | | | | |
| S | *L. nubigenus* Kunth | 25.0 | i | w | | | | | |
| I | *L. odoratus* A.Heller | 22.0 | n | h | | | | | |
| O | *L. pachylobus* Greene | 27.0 | n | h | | | | | |
| Q | *L. perennis* L. | 50.0 | n | w | | | | | |
| S | *L. piurensis* C.P.Sm. | 55.0 | i | h | | | | | |
| P | *L. polyphyllus* Lindl. | 67.0 | i | w | | | | | |
| S | *L. praestabilis* C.P.Sm. | 225.0 | i | w | | | | | |
| S | *L. prostratus* J.Agardh | 12.0 | i | w | | | | | |
| S | *L. pubescens* Benth. | 100.0 | i | w | | | | | |

*continued*

| Clade | Taxon | Plant height | Distri-bution | Life history | Molecular marker ITS | D6DES | rbcL | trnL | trnLF |
|---|---|---|---|---|---|---|---|---|---|
| S | *L. pulvinaris* Ulbr. | 2.0 | i | w | | | | | |
| S | *L. purosericeus* C.P.Sm. | 75.0 | i | w | | | | | |
| S | *L. ramosissimus* Benth. | 160.0 | i | w | | | | | |
| P | *L. rivularis* Lindl. | 65.0 | i | w | | | | | |
| S | *L. sarmentosus* Desr. | 15.0 | i | w | | | | | |
| S | *L. semperflorens* Benth. | 550.0 | i | w | | | | | |
| P | *L. sericeus* Pursh | 90.0 | i | w | | | | | |
| I | *L. shockleyi* S.Watson | 12.0 | n | h | | | | | |
| P | *L. sierrae-blancae* Wooton & Standl. | 100.0 | i | w | | | | | |
| S | *L. solanagrorum* C.P. Sm. | 170.0 | i | w | | | | | |
| K | *L. sparsiflorus* Benth. | 27.0 | n | h | | | | | |
| M | *L. stiversii* Kellogg | 30.0 | n | h | | | | | |
| S | *L. subacaulis* Griseb. | 12.0 | i | w | | | | | |
| L | *L. succulentus* K.Koch | 45.0 | n | h | | | | | |
| P | *L. sulphureus* Hook. | 60.0 | i | w | | | | | |
| S | *L. tarapacensis* C.P.Sm. | 85.0 | i | w | | | | | |
| P | *L. tegeticulatus* Eastw. | 8.0 | i | w | | | | | |
| S | *L. tomentosus* DC. | 170.0 | i | w | | | | | |
| S | *L. tominensis* Wedd. | 80.0 | i | w | | | | | |
| K | *L. truncatus* Hook. & Arn. | 30.0 | n | h | | | | | |
| I | *L. uncialis* S.Watson | 1.5 | n | h | | | | | |
| S | *L. weberbaueri* Ulbr. | 160.0 | i | w | | | | | |
| **Silverswords—Tarweeds (Madiinae)** | | | | | | | | | |
| E | *Achyrachaena mollis* Schauer | 33.0 | n | h | AF229318 | | | | |
| E | *Adenothamnus validus* (Brandegee) D.D.Keck | 17.5 | n | w | M93787 | | | | |
| O | *Anisocarpus madioides* Nutt. | 47.5 | n | h | AF061914 | | | | |
| O | *A. scabridus* (Eastw.) B.G.Baldwin | 30.0 | n | h | M93799 | | | | |
| P | *Argyroxiphium caliginis* C.N.Forbes | 84.5 | i (2) | w | M93788 | | | | |
| P | *A. grayanum* (Hillebr.) O.Deg. | 199.9 | i (2) | w | AF061885 | | | | |
| P | *A. kauense* (Rock & Neal) O.Deg. & I.Deg. | 160.0 | i (2) | w | AF061887 | | | | |
| P | *A. sandwicense* DC. | 300.1 | i (2) | w | EU341969 | | | | |
| A | *Blepharipappus scaber* Hook. | 12.5 | n | h | AF229316 | | | | |
| I | *Blepharizonia laxa* Greene | 95.0 | n | h | AF283548 | | | | |
| I | *B. plumosa* (Kellogg) Greene | 95.0 | n | h | AF283551 | | | | |
| G | *Calycadenia fremontii* A.Gray | 55.0 | n | h | U04249 | | | | |
| G | *C. hooveri* G.D.Carr | 35.0 | n | h | U04251 | | | | |

*continued*

| Clade | Taxon | Plant height | Distri-bution | Life history | Molecular marker | | | | |
|---|---|---|---|---|---|---|---|---|---|
| | | | | | **ITS** | **D6DES** | **rbcL** | **trnL** | **trnLF** |
| G | *C. mollis* A.Gray | 39.0 | n | h | U04253 | | | | |
| G | *C. multiglandulosa* DC. | 40.0 | n | h | U04254 | | | | |
| G | *C. oppositifolia* (Greene) Greene | 20.0 | n | h | U04256 | | | | |
| G | *C. pauciflora* A.Gray | 25.0 | n | h | U04259 | | | | |
| G | *C. spicata* (Greene) Greene | 40.0 | n | h | U04260 | | | | |
| G | *C. truncata* DC. | 70.0 | n | h | U04262 | | | | |
| G | *C. villosa* DC. | 27.5 | n | h | U04263 | | | | |
| J | *Carlquistia muirii* (A.Gray) B.G.Baldwin | 30.5 | n | h | M93798 | | | | |
| H | *Centromadia perennis* Greene | 87.5 | n | h | U04265 | | | | |
| N | *Deinandra arida* (D.D.Keck) B.G.Baldwin | 50.0 | n | h | EF059610 | | | | |
| N | *D. bacigalupii* B.G.Baldwin | 25.0 | n | h | EF059701 | | | | |
| N | *D. clementina* (Brandegee) B.G.Baldwin | 55.0 | i (1) | w | EF059624 | | | | |
| L | *D. conjugens* (D.D.Keck) B.G.Baldwin | 30.0 | n | h | EF059606 | | | | |
| N | *D. corymbosa* (DC.) B.G.Baldwin | 53.0 | n | h | EF059691 | | | | |
| L | *D. fasciculata* (DC.) Greene | 52.0 | n | h | EF059605 | | | | |
| N | *D. floribunda* (A.Gray) Davidson & Moxley | 65.0 | n | h | EF059608 | | | | |
| N | *D. frutescens* (A.Gray) B.G.Baldwin | 40.0 | i (1) | w | EF059660 | | | | |
| N | *D. greeneana* (Rose) B.G.Baldwin | 75.0 | i (1) | w | EF059644 | | | | |
| N | *D. halliana* (D.D.Keck) B.G.Baldwin | 67.5 | n | h | EF059697 | | | | |
| N | *D. increscens* (H.M.Hall ex D.D.Keck) B.G.Baldwin | 52.0 | n | h | EF059688 | | | | |
| N | *D. kellogii* Greene | 37.5 | n | h | EF059692 | | | | |
| N | *D. lobbii* (Greene) Greene | 37.5 | n | h | EF059700 | | | | |
| N | *D. martirensis* (D.D.Keck) B.G.Baldwin | 80.0 | n | w | EF059643 | | | | |
| N | *D. minthornii* (Jeps.) B.G.Baldwin | 57.5 | n | w | EF059612 | | | | |
| N | *D. mohavensis* (D.D.Keck) B.G.Baldwin | 55.0 | n | h | EF059685 | | | | |
| N | *D. pallida* (D.D.Keck) B.G.Baldwin | 54.5 | n | h | EF059694 | | | | |

*continued*

| Clade | Taxon | Plant height | Distri-bution | Life history | Molecular marker ITS | D6DES | rbcL | trnL | trnLF |
|---|---|---|---|---|---|---|---|---|---|
| N | *D. palmeri* (Rose) B.G.Baldwin | 10.0 | i (1) | w | EF059651 | | | | |
| N | *D. paniculata* (A.Gray) Davidson & Moxley | 45.0 | n | h | EF059687 | | | | |
| N | *D. pentactis* (D.D.Keck) B.G.Baldwin | 39.5 | n | h | EF059699 | | | | |
| N | *D. streetsii* (A.Gray) B.G.Baldwin | 20.0 | i (1) | w | EF059625 | | | | |
| Q | *Dubautia arborea* (A.Gray) D.D.Keck | 600.0 | i (2) | w | EU341945 | | | | |
| Q | *D. ciliolata* (DC.) D.D.Keck | 180.0 | i (2) | w | AF061892 | | | | |
| Q | *D. herbstobatae* G.D.Carr | 50.0 | i (2) | w | AF061901 | | | | |
| R | *D. imbricata* H.St.John & G.D.Carr | 249.9 | i (2) | w | AF061912 | | | | |
| R | *D. laevigata* A.Gray | 249.9 | i (2) | w | AF061898 | | | | |
| R | *D. laxa* Hook. & Arn. | 500.2 | i (2) | w | AF061893 | | | | |
| Q | *D. linearis* (Gaudich.) D.D.Keck | 300.1 | i (2) | w | AF061910 | | | | |
| Q | *D. menziesii* (A.Gray) D.D.Keck | 249.9 | i (2) | w | EU341949 | | | | |
| R | *D. microcephala* Skottsb. | 399.8 | i (2) | w | AF061902 | | | | |
| R | *D. paleata* A.Gray | 140.1 | i (2) | w | AF061888 | | | | |
| R | *D. pauciflorula* H.St.John & G.D.Carr | 300.1 | i (2) | w | AF061896 | | | | |
| R | *D. plantaginea* Gaudich. | 699.9 | i (2) | w | AF061889 | | | | |
| Q | *D. platyphylla* (A.Gray) D.D.Keck | 350.0 | i (2) | w | AF061908 | | | | |
| R | *D. raillardioides* Hillebr. | 300.1 | i (2) | w | AF061897 | | | | |
| Q | *D. reticulata* (Sherff) D.D.Keck | 800.3 | i (2) | w | AF061895 | | | | |
| Q | *D. scabra* (DC.) D.D.Keck | 25.0 | i (2) | w | AF061906 | | | | |
| Q | *D. sherffiana* Fosberg | 199.9 | i (2) | w | AF061907 | | | | |
| Q | *D. waianapanapaensis* G.D.Carr | 30.0 | i (2) | w | EU341967 | | | | |
| F | *Hemizonella minima* (A.Gray) A.Gray | 10.5 | n | h | AF229317 | | | | |
| F | *H. congesta* DC. | 42.5 | n | h | AF283544 | | | | |
| M | *Holocarpha heermannii* (Greene) D.D.Keck | 65.0 | n | h | EF059603 | | | | |
| M | *H. virgata* (A.Gray) D.D.Keck | 70.0 | n | h | AF229321 | | | | |
| F | *Holozonia filipes* (Hook. & Arn.) Greene | 90.0 | n | h | AF229312 | | | | |
| F | *Kyhosia bolanderi* (A.Gray) B.G.Baldwin | 85.0 | n | h | M93794 | | | | |

*continued*

| Clade | Taxon | Plant height | Distri-bution | Life history | Molecular marker ITS | D6DES | rbcL | trnL | trnLF |
|---|---|---|---|---|---|---|---|---|---|
| B | *Lagophylla glandulosa* A.Gray | 55.0 | n | h | DQ188073 | | | | |
| B | *L. minor* (D.D.Keck) D.D.Keck | 19.0 | n | h | AF229311 | | | | |
| B | *L. ramosissima* Nutt. | 55.0 | n | h | AF229310 | | | | |
| C | *Layia carnosa* (Nutt.) Torr. & A.Gray | 10.0 | n | h | DQ188045 | | | | |
| C | *L. chrysanthemoides* (DC. ex DC.) A.Gray | 28.5 | n | h | DQ188077 | | | | |
| C | *L. discoidea* D.D.Keck | 11.5 | n | h | DQ188068 | | | | |
| C | *L. gaillardioides* (Hook. & Arn.) DC. | 33.0 | n | h | DQ188044 | | | | |
| C | *L. glandulosa* (Hook.) Hook. & Arn. | 31.5 | n | h | DQ188063 | | | | |
| C | *L. heterotricha* (DC.) Hook. & Arn. | 51.5 | n | h | DQ188075 | | | | |
| C | *L. munzii* D.D.Keck | 28.0 | n | h | AF229314 | | | | |
| C | *L. pentachaeta* A.Gray | 52.5 | n | h | DQ188046 | | | | |
| C | *L. septentrionalis* D.D.Keck | 20.5 | n | h | DQ188080 | | | | |
| K | *Madia elegans* D.Don ex Lindl. | 128.0 | n | h | AF413612 | | | | |
| K | *M. sativa* Molina | 67.5 | n | h | EU853464 | | | | |
| F | *Osmadenia tenella* Nutt. | 22.5 | n | h | U04266 | | | | |
| D | *Raillardella argentea* (A.Gray) A.Gray | 8.0 | n | h | AF229309 | | | | |
| D | *R. pringlei* Greene | 37.5 | n | h | M93797 | | | | |
| R | *Wilkesia gymnoxiphium* A.Gray | 300.1 | i (2) | w | M93800 | | | | |
| R | *W. hobdyi* H.St.John | 70.0 | i (2) | w | AF061882 | | | | |

Distribution: n, non-island (mainland or lowland); i, island; i (1), California islands; i (2), Hawaiian archipelago;
Life history: w, woody (= perennial in *Lupinus*); h, herbaceous (= annual in *Lupinus*);
Plant height, mean plant height in cm.

**Table S2** Comparative diversification rate analysis detailing model specification and model fit used in Bayesian model averaging (BayesRate).

| | Model | | | | | | | | |
|---|---|---|---|---|---|---|---|---|---|
| Clade | No | Non-island | (Sky-) Island | Island 2 | Clades linked | MlnL | BF | *p* | # samples |
| ***Echium*** | | | | | | | | | |
| | M1 | Yule | Yule | — | No | -27.64 | 0.00 | 0.471 | 990 |
| | M3 | Yule | birth-death | — | No | -28.46 | 1.64 | 0.208 | 437 |
| | M5 | birth-death | Yule | — | No | -29.03 | 2.77 | 0.118 | 247 |
| | M7 | birth-death | birth-death | — | No | -29.50 | 3.71 | 0.074 | 155 |
| | M4 | Yule | birth-death | — | Yes | -29.94 | 4.60 | 0.047 | 99 |
| | M8 | birth-death | birth-death | — | Yes | -29.97 | 4.65 | 0.046 | 97 |
| | M2 | Yule | Yule | — | Yes | -30.27 | 5.26 | 0.034 | 72 |
| | M6 | birth-death | Yule | — | Yes | -32.57 | 9.86 | 0.003 | 7 |
| ***Hypericum*** | | | | | | | | | |
| | M5 | birth-death | Yule | — | No | -151.29 | 0.00 | 0.823 | 990 |
| | M7 | birth-death | birth-death | — | No | -152.86 | 3.13 | 0.172 | 207 |
| | M1 | Yule | Yule | — | No | -156.61 | 10.63 | 0.004 | 5 |
| | M3 | Yule | birth-death | — | No | -157.82 | 13.05 | 0.001 | 1 |
| | M8 | birth-death | birth-death | — | Yes | -163.39 | 24.19 | 0.000 | 0 |
| | M2 | Yule | Yule | — | Yes | -177.50 | 52.42 | 0.000 | 0 |
| | M4 | Yule | birth-death | — | Yes | -177.56 | 52.53 | 0.000 | 0 |
| | M6 | birth-death | Yule | — | Yes | -179.71 | 56.82 | 0.000 | 0 |
| ***Lupinus*** | | | | | | | | | |
| | M1 | Yule | Yule | — | No | -120.77 | 0.00 | 0.523 | 990 |
| | M3 | Yule | birth-death | — | No | -121.30 | 1.06 | 0.308 | 583 |
| | M5 | birth-death | Yule | — | No | -122.33 | 3.11 | 0.111 | 209 |
| | M7 | birth-death | birth-death | — | No | -122.96 | 4.37 | 0.059 | 112 |
| | M8 | birth-death | birth-death | — | Yes | -147.89 | 54.23 | 0.000 | 0 |
| | M4 | Yule | birth-death | — | Yes | -155.41 | 69.27 | 0.000 | 0 |
| | M2 | Yule | Yule | — | Yes | -155.51 | 69.48 | 0.000 | 0 |
| | M6 | birth-death | Yule | — | Yes | -158.57 | 75.59 | 0.000 | 0 |
| **Silverswords & Tarweeds (Madiinae)** | | | | | | | | | |
| | M28 | Yule | birth-death | Yule | Hwi. unl. | -181.39 | 0.00 | 0.148 | 990 |
| | M22 | Yule | Yule | b-d | Isls. link. | -181.41 | 0.03 | 0.146 | 974 |
| | M17 | Yule | birth-death | b-d | No | -181.55 | 0.33 | 0.126 | 841 |
| | M2 | Yule | Yule | Yule | Isls. link. | -181.57 | 0.36 | 0.124 | 825 |
| | M14 | birth-death | birth-death | b-d | Isls. link. | -181.90 | 1.02 | 0.089 | 595 |
| | M26 | Yule | birth-death | Yule | Isls. link. | -181.98 | 1.17 | 0.083 | 551 |
| | M27 | Yule | birth-death | Yule | Yes | -182.08 | 1.38 | 0.074 | 496 |
| | M20 | Yule | birth-death | b-d | Yes | -182.37 | 1.96 | 0.056 | 372 |
| | M24 | Yule | Yule | b-d | Yes | -182.87 | 2.96 | 0.034 | 225 |
| | M5 | birth-death | Yule | Yule | No | -182.92 | 3.07 | 0.032 | 213 |
| | M23 | Yule | Yule | b-d | Hwi. unl. | -183.08 | 3.38 | 0.027 | 183 |
| | M25 | Yule | birth-death | Yule | No | -183.52 | 4.27 | 0.018 | 117 |
| | M8 | birth-death | Yule | Yule | Yes | -183.94 | 5.10 | 0.012 | 77 |
| | M1 | Yule | Yule | Yule | No | -184.20 | 5.62 | 0.009 | 60 |
| | M11 | birth-death | birth-death | Yule | Hwi. unl. | -184.27 | 5.76 | 0.008 | 55 |
| | M16 | birth-death | birth-death | b-d | Yes | -184.75 | 6.73 | 0.005 | 34 |
| | M19 | Yule | birth-death | b-d | Hwi. unl. | -184.93 | 7.09 | 0.004 | 29 |
| | M13 | birth-death | birth-death | b-d | No | -185.44 | 8.09 | 0.003 | 17 |
| | M4 | Yule | Yule | Yule | Yes | -185.76 | 8.74 | 0.002 | 13 |
| | M7 | birth-death | Yule | Yule | Hwi. unl. | -186.76 | 10.74 | 0.001 | 5 |
| | M10 | birth-death | birth-death | Yule | Isls. link. | -187.19 | 11.61 | 0.000 | 3 |
| | M18 | Yule | birth-death | b-d | Isls. link. | -188.15 | 13.53 | 0.000 | 1 |
| | M12 | birth-death | birth-death | Yule | Yes | -188.26 | 13.75 | 0.000 | 1 |

*continued*

| Clade | Model | | | | | MlnL | BF | *p* | # samples |
|---|---|---|---|---|---|---|---|---|---|
| | No | Non-island | (Sky-) Island | Island 2 | Clades linked | | | | |
| | M15 | birth-death | birth-death | b-d | Hwi. unl. | -188.27 | 13.75 | 0.000 | 1 |
| | M3 | Yule | Yule | Yule | Hwi. unl. | -188.34 | 13.90 | 0.000 | 1 |
| | M21 | Yule | Yule | b-d | No | -188.39 | 14.00 | 0.000 | 1 |
| | M9 | birth-death | birth-death | Yule | No | -190.71 | 18.63 | 0.000 | 0 |
| | M6 | birth-death | Yule | Yule | Isls. link. | -191.08 | 19.38 | 0.000 | 0 |

MlnL, natural log marginal likelihood, BF, Bayes factor; *p*, relative probability; # samples, number of samples included in the combined posterior.
Madiinae: Island, Californian Islands; Island 2, Hawaiian Islands.
Yule, pure birth model; b-d, birth-death model; Hwi. unl., Hawaiian silverswords clade not linked, i.e. non-island and Californian islands clade linked; Isls. link., both island clades linked among each other and unlinked with non-island species.

**Table S3** Sampling fractions used to account for incomplete species sampling in the exploratory diversification rate heterogeneity analysis (BAMM).

| Clade | *Echium* | *Hypericum* | *Lupinus* | Silverswords-Tarweeds |
|---|---|---|---|---|
| A | 1.00 | 0.67 | — | 1.00 |
| B | 0.60 | 0.90 | — | 0.75 |
| C | 1.00 | 0.35 | — | 0.64 |
| D | 0.65 | 0.36 | — | 0.67 |
| E | 1.00 | 1.00 | — | 1.00 |
| F | — | 0.33 | — | 1.00 |
| G | — | 0.50 | — | 0.90 |
| H | — | 0.37 | — | 0.25 |
| I | — | 0.63 | 0.70 | 1.00 |
| J | — | — | 1.00 | 1.00 |
| K | — | — | 0.83 | 0.20 |
| L | — | — | 1.00 | 1.00 |
| M | — | — | 1.00 | 0.50 |
| N | — | — | 1.00 | 1.00 |
| O | — | — | 0.50 | 1.00 |
| P | — | — | 0.41 | 0.80 |
| Q | — | — | 0.60 | 0.86 |
| R | — | — | 0.13 | 1.00 |
| S | — | — | 0.38 | — |

*Lupinus* clade names follow Drummond et al. (2012b) with clades A–H pruned in our tree.
For clade assignment see Figs. S1–S4.
See main article Table 1 for sampling fractions used in Bayesian model averaging (BayesRate).

**Table S4.** Plant height: Continuous trait models of evolution ranked according to model fit per study group (OUwie).

| Clade | Model | df | lnL | AICc | ΔAICc | Akaike weight | Eigenvalues negative | Model failure |
|---|---|---|---|---|---|---|---|---|
| ***Echium*** | | | | | | | | |
| | OUθ | 4 | -38.301 | 85.533 | 0 | 0.6111 | 0 | 0 |
| | OUθ$\sigma^2$ | 5 | -37.856 | 87.141 | 1.609 | 0.2734 | 0 | 0 |
| | OU1 | 3 | -41.737 | 90.019 | 4.486 | 0.0649 | 0 | 0 |
| | BM$\sigma^2$ | 4 | -40.791 | 90.512 | 4.979 | 0.0507 | 0 | 0 |
| | BM1 | 2 | -55.846 | 115.959 | 30.426 | 1.5108e-07 | 0 | 0 |
| | OUθα | 5 | NA | NA | NA | NA | 85% | 100% |
| ***Hypericum*** | | | | | | | | |
| | OU1 | 3 | -109.096 | 224.432 | 0 | 0.3339 | 0 | 0 |
| | OUθ | 4 | -108.294 | 224.992 | 0.56 | 0.2524 | 0 | 0 |
| | OUθ$\sigma^2$ | 5 | -107.363 | 225.338 | 0.906 | 0.2123 | 0 | 0 |
| | OUθα | 5 | -107.416 | 225.444 | 1.011 | 0.2014 | 52% | 90% |
| | BM$\sigma^2$ | 4 | -128.922 | 266.248 | 41.816 | 2.7768e-10 | 0 | 0 |
| | BM1 | 2 | -135.665 | 275.449 | 51.017 | 2.7887e-12 | 0 | 0 |
| ***Lupinus*** | | | | | | | | |
| | OUθ | 4 | -118.864 | 246.234 | 0 | 0.5686 | 0 | 0 |
| | OUθ$\sigma^2$ | 5 | -118.663 | 248.095 | 1.861 | 0.2242 | 0 | 0 |
| | OUθα | 5 | -118.759 | 248.288 | 2.054 | 0.2036 | 75% | 99% |
| | OU1 | 3 | -125.021 | 256.341 | 10.107 | 0.0036 | 0 | 0 |
| | BM$\sigma^2$ | 4 | -131.172 | 270.851 | 24.617 | 2.5658e-6 | 0 | 0 |
| | BM1 | 2 | -143.347 | 290.842 | 44.608 | 1.1704e-10 | 0 | 0 |
| **Silverswords—Tarweeds (Madiinae)** | | | | | | | | |
| | OUθ$\sigma^2$ | 7 | -89.499 | 194.415 | 0 | 0.7172 | 24% | 0 |
| | OUθ | 5 | -92.768 | 196.276 | 1.861 | 0.2828 | 99% | 0 |
| | OU1 | 3 | -126.612 | 259.513 | 65.098 | 5.2452e-15 | 0 | 0 |
| | BM$\sigma^2$ | 6 | -130.446 | 273.943 | 79.528 | 3.8578e-18 | 0 | 0 |
| | BM1 | 2 | -176.163 | 356.469 | 162.054 | 4.6349e-36 | 0 | 0 |
| | OUθα | 7 | NA | NA | NA | NA | 100% | 100% |

df, degree of freedom; lnL, natural log likelihood; AICc, sample size corrected Akaike information criterium; ΔAICc, difference in AICc to the best model tested; Eigenvalues negative' and 'Model failure', model performance indices (note that the results of the runs with either negative eigenvalues or failed models were excluded before model averaging).

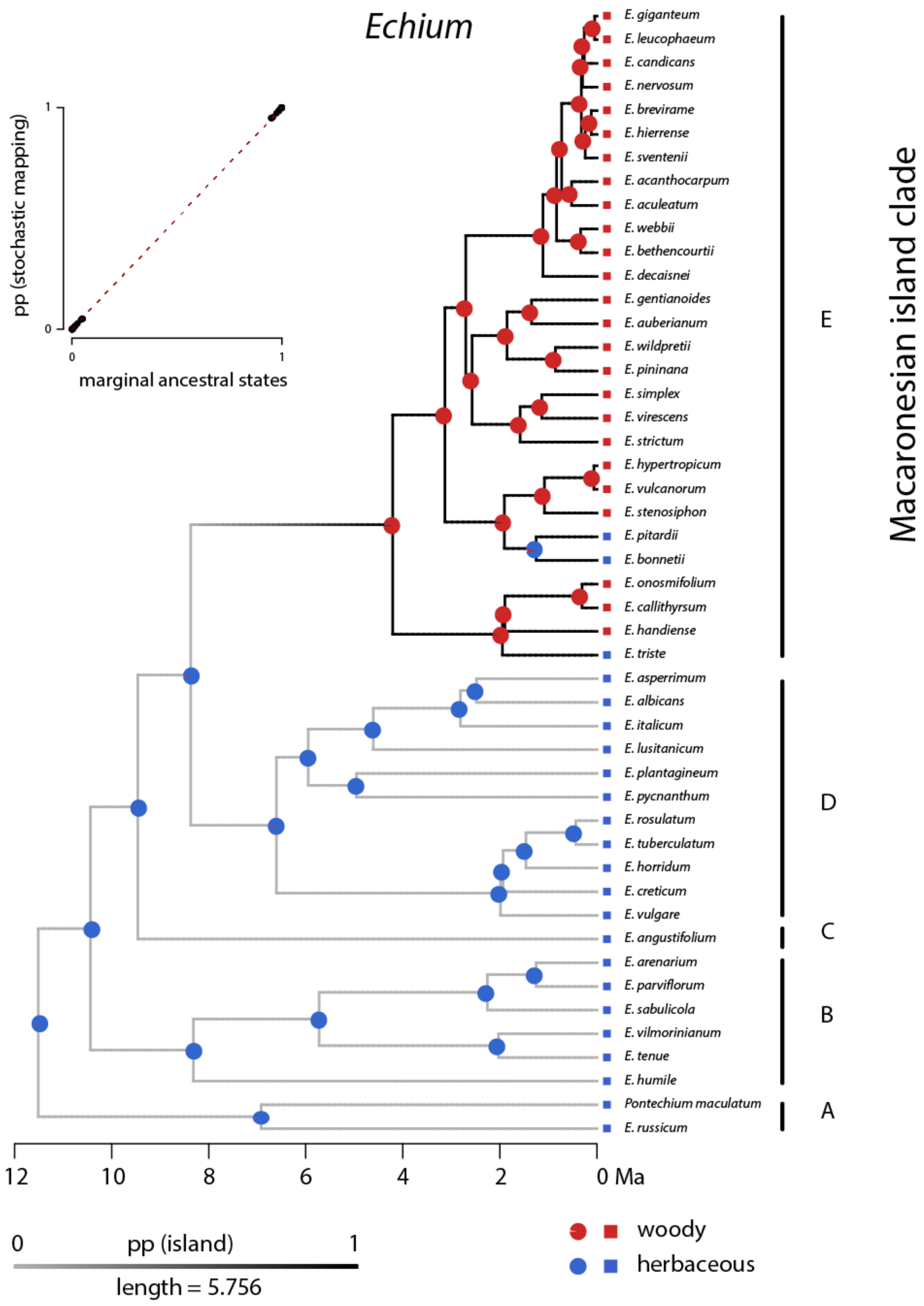


**Fig. S1.** Ancestral state estimates in *Echium* of life history evolution (tip and node labels, obtained by stochastic character mapping (SCM) of life history strategy) overlaid on a stochastic mapping of occurrence (island / non-island, gradient on branches). The insert top left compares results of SCM (*make.simmap*) to ML (*ace*) estimates of life history. Clade names correspond to tables S1, S4.

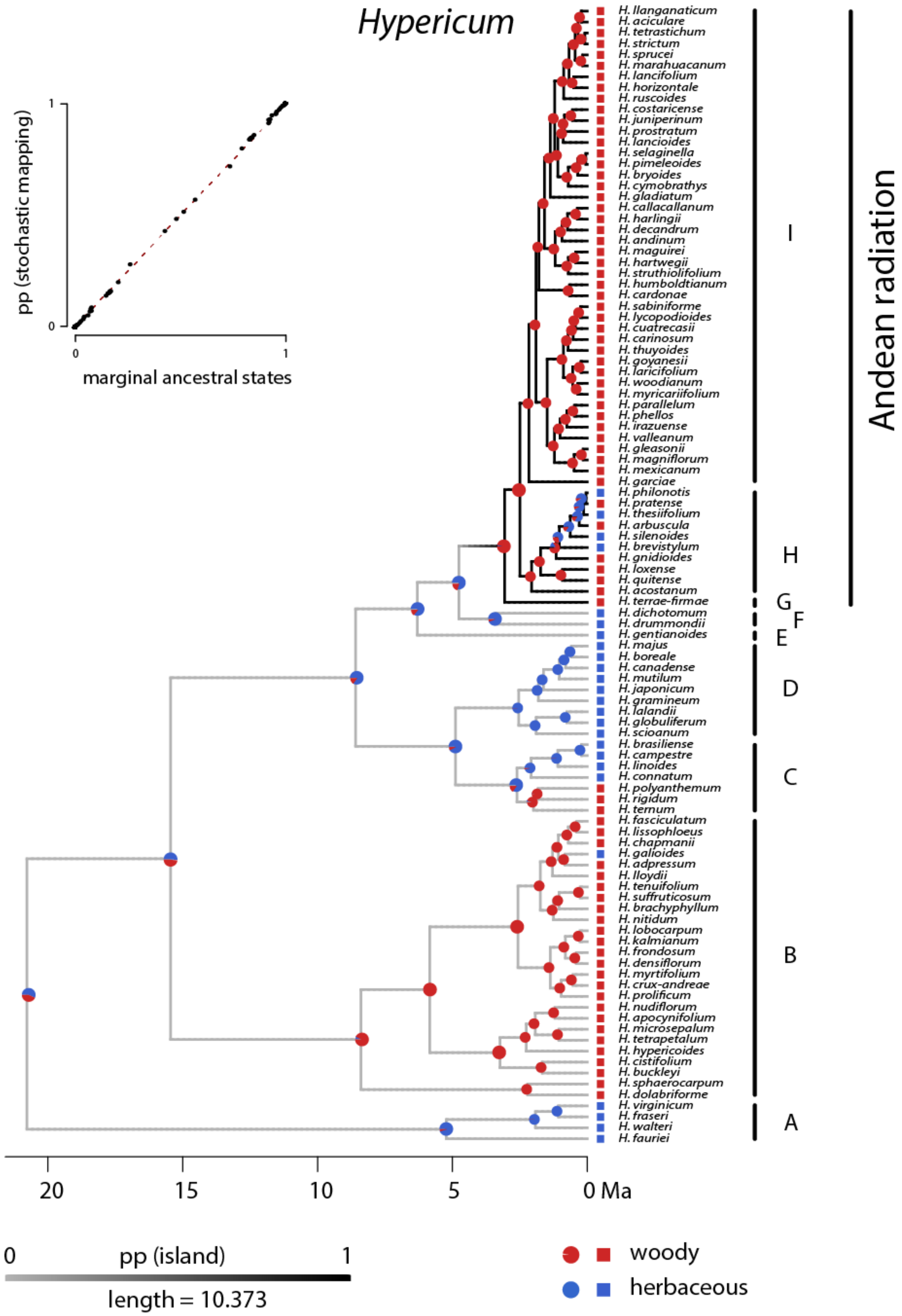


**Fig. S2.** Ancestral state estimates in *Hypericum* of life history evolution (tip and node labels, obtained by stochastic character mapping (SCM) of life history strategy) overlaid on a stochastic mapping of occurrence (island / non-island, gradient on branches). The insert top left compares results of SCM (*make.simmap*) to ML (*ace*) estimates of life history. Clade names correspond to tables S1, S4.

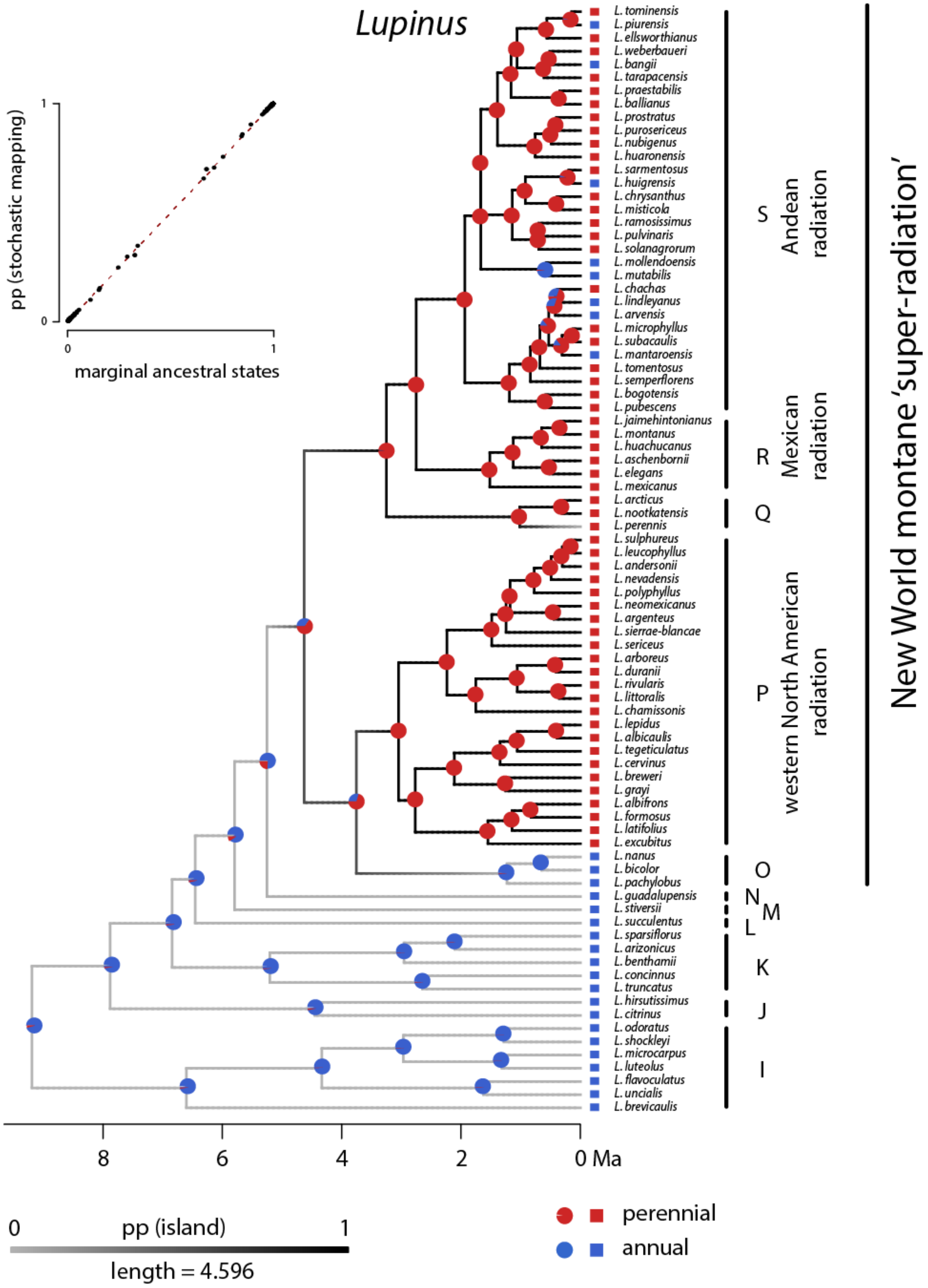


**Fig. S3.** Ancestral state estimates in *Lupinus* of life history evolution (tip and node labels, obtained by stochastic character mapping (SCM) of life history strategy) overlaid on a stochastic mapping of occurrence (island / non-island, gradient on branches). The insert top left compares results of SCM (*make.simmap*) to ML (*ace*) estimates of life history. Clade names correspond to tables S1, S4.

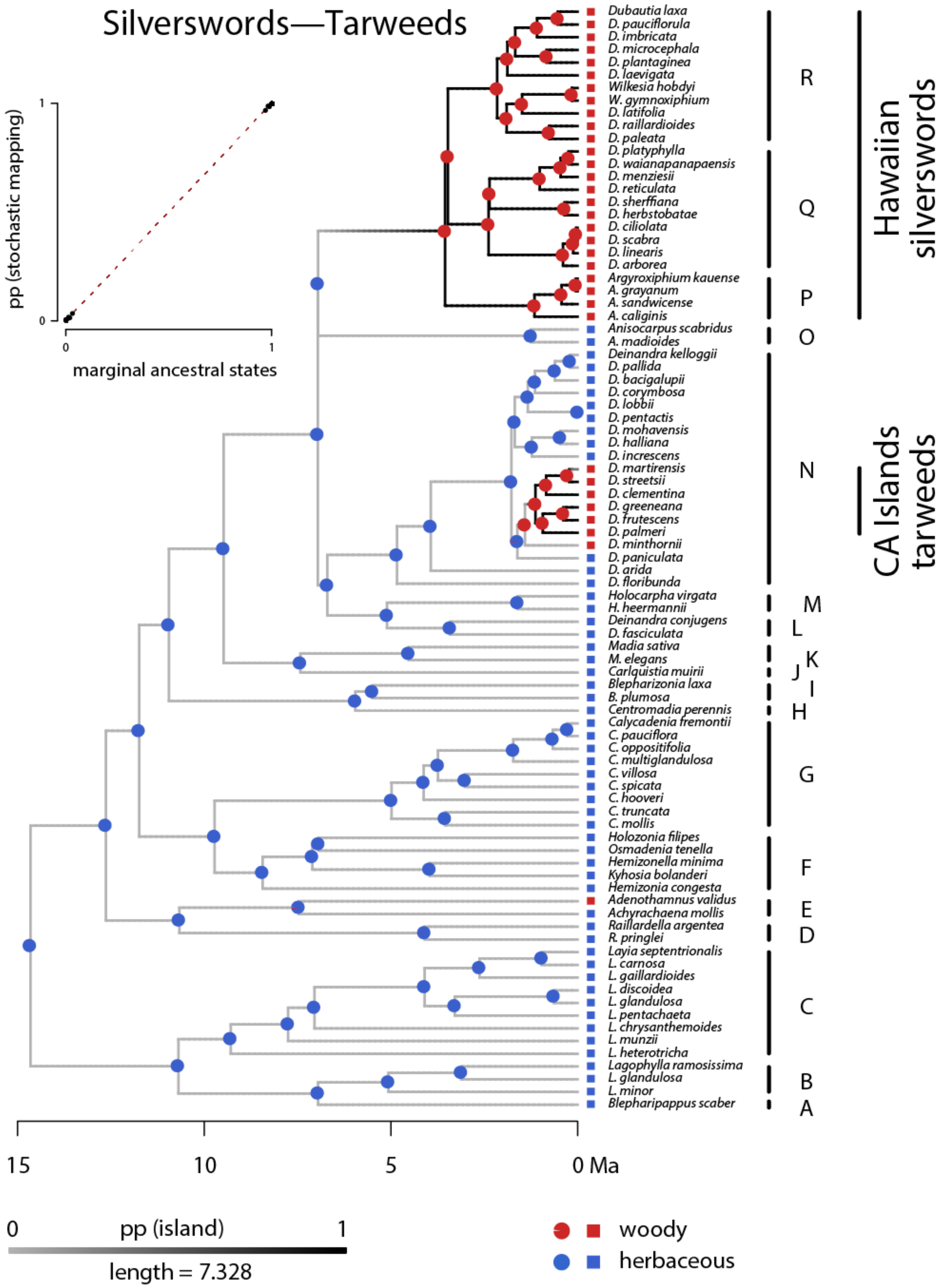


**Fig. S4.** Ancestral state estimates in the Silverswords—Tarweeds of life history evolution (tip and node labels, obtained by stochastic character mapping (SCM) of life history strategy) overlaid on a stochastic mapping of occurrence (island / non-island, gradient on branches). The insert top left compares results of SCM (*make.simmap*) to ML (*ace*) estimates of life history. Clade names correspond to tables S1, S4.

**Methods S1** Clade specific information, phylogenetic reconstructions and trait data

All data used in this study (DNA alignments, xml BEAST input files, phylogenetic trees, trait data tables) are available on Dryad (https://doi.org/10.5061/dryad.rt530k9).

***Echium*** – The genus *Echium* L. comprises ca. 68 species native to North Africa, mainland Europe and the Macaronesian islands. Early phylogenetic work demonstrated the monophyly of the Macaronesian species and characterised this island radiation as a classical example of insular woodiness and disparification of insular growth forms (Böhle *et al.*, 1996). More recently published phylogenies of *Echium* either contain too few non-island taxa (García-Maroto *et al.*, 2009) or lack a timeline (Romeiras *et al.*, 2011). In order to provide a time-calibrated comparative framework encompassing the island and mainland diversity for evolutionary analyses we generated a new time-calibrated phylogenetic tree for all major lineages of Boraginaceae with as complete sampling as possible of the genus *Echium*, using data for two chloroplast markers (rbcL and trnLF) and two nuclear markers (ITS and D6DES) downloaded from GenBank (GenBank accession numbers are provided in table S1). Sequences were aligned using MUSCLE v3.6 (Edgar, 2004) and alignments were visually crosschecked and ambiguous fragments were excluded using Geneious v6.0.5 (Kearse *et al.*, 2011). To test on supported topological conflict between the individual gene trees phylogenies were inferred for each individual marker using RAxML v8 (Stamatakis, 2006) with a GTR + Γ substitution model and rapid bootstrapping with 100 iterations. Since no supported conflicts were present, we concatenated the alignments prior to estimating the topology and divergence times simultaneously using BEAST v1.8.2 (Drummond, AJ *et al.*, 2012) under an uncorrelated lognormal relaxed-clock model, using the general time-reversible (GTR) substitution rate model and Γ-distributed rates among sites with a proportion of invariant sites to describe the rate heterogeneity among sites, with a Yule model as tree prior. We used critically selected fossils for calibration: the genus *Ehretia* P.Browne has a good fossil record from the early Eocene to the Neogene and represents the earliest fossil record of Boraginaceae, therefore we used (1) this set of fossils to set a minimum age of 33.9 Myr (Chandler, 1962; Mai & Walther, 1991; Ozaki, 1991). Other fossils used were: (2) a fossil of *Cryptantha* Lehm. ex G.Don (Darnell & Thomasson, 2007), and (3) a fossil of *Lithospermum* L. (Gabel, 1987), both from the late Miocene Ash Hollow Formation in the Ogallala group from c. 10.3 to c. 13.6 Ma; (4) a fossil nutlet and pollen records of *Tournefortia* L. from the Oligocene (Dorofeev, 1963); and (5) a fossil of *Cordia* L. from the Eocene (Huzioka & Takahasi, 1970). The fossils were assigned to clades on the basis of their morphology and the respective nodes were constrained with uniform priors with the minimum age set by the age of the fossil, and the maximum age of 88 Myr. We ran four independent Markov Chain Monte Carlo (MCMC) runs of $50 \times 10^6$ generations and checked for convergence between runs, and effective sample sizes of >200 for

all parameters in Tracer 1.5 (Rambaut & Drummond, 2007). We combined the four runs in LogCombiner after discarding the first 10% generations (burnin) in each run and we selected and annotated the Maximum Clade Credibility (MCC) tree using TreeAnnotator. The MCC tree was used in all subsequent comparative analyses after pruning the outgroup taxa.

In our time-calibrated phylogeny of *Echium* all Macaronesian taxa form a monophyletic group, concordant with the topologies of Böhle *et al.* (1996) and García-Maroto *et al.* (2009), with all three Macaronesian clades recovered by the latter also recovered in our own phylogeny (Fig. S1). Our crown age estimate for the Macaronesian clade is slightly younger, ca. 4.2 Ma vs 6.9 Ma, however, the age estimate recovered by García-Maroto *et al.* (2009) falls within our 95% HPD. Our phylogeny also recovers the Cape Verde Islands relationship of *E. stenosiphon* as sister to *E. hypertropicum* + *E. vulcanorum* as presented by Romeiras *et al.* (2011).

Trait measurements were obtained from the *Echium* account from Flora Iberica (Talavera *et al.*, 2012) and Bramwell's (1972) account of Macaronesian *Echium*.

***Hypericum*** – While the main centre of species diversity for *Hypericum* L. with around 330 species is in the Old World (Nürk *et al.*, 2015), ca. 170 species occur in the Americas, thereof 120 in Latin America, of which ca. 70 are Andean páramo endemics (NB ca. 99 species in the 'Andean radiation' [clades G, H, I; Fig. S2] due to 'secondary out-of-páramo' dispersals; Nürk *et al.*, 2018), with the 'sky island' here defined as the high elevation páramo ecosystem above the treeline (Nürk *et al.*, 2018). To ensure as complete sampling as possible for the páramo endemics and related taxa, we used a previously published time-calibrated phylogeny, which has extensive sampling of the New World lineages (Nürk *et al.*, 2018). Previous work has demonstrated a close association between arborescence and tropical montane occurrences in African mountains and the Andes (Nürk *et al.*, 2013a) and elevated species richness associated with the very recent Pliocene / Quaternary radiation in the Andes, a radiation encompassing a disparate array of growth forms typical of tropical alpine sky island plant groups (Nürk *et al.*, 2013b).

Trait measurements were obtained from cladistic studies (Nürk & Blattner, 2010), herbarium specimen data, and the monograph of *Hypericum* (Robson, 1987; Robson, 1990; Robson, 1996; Robson, 2012).

***Lupinus*** – The legume genus *Lupinus* L. comprises ca. 290 species, of which ca. 190 occur across Mexico to South America, thereof ca. 85 across the Andes mountain range (Hughes & Eastwood, 2006; Drummond, CS *et al.*, 2012; Nevado *et al.*, 2016). We used the best currently available time-calibrated phylogeny (Drummond, CS *et al.*, 2012), pruned to the robustly supported large western New World clade containing c. 220 species found in western North and South America

(i.e. clades I to S of Drummond et al., 2012). Previous work demonstrated significant associations between shifts from annual to perennial life histories, lowland to montane habitats and accelerated rates of species diversification nested within this clade, with these three coincident shifts subtending a large western New World montane 'super-radiation' within which is nested the Andean radiation (Drummond, CS *et al.*, 2012). Like the Andean radiation of *Hypericum*, Andean *Lupinus* also includes diverse growth forms (Hughes & Eastwood, 2006; Hughes & Atchison, 2015). For *Lupinus* the western New World 'super-radiation' *sensu* Drummond, CS *et al.* (2012) and Hughes and Atchison (2015) is defined as the montane sky island clade and the set of lowland western North American annual species that subtend the montane radiations are the non-island relatives.

Trait measurements were obtained from field observations, herbarium specimen data and literature (Gladstones, 1974; McVaugh & Anderson, 1987; Barneby, 1989; Baldwin *et al.*, 2012).

**Silverswords–Tarweeds** – The subtribe Madiinae Benth. & Hook.f. (Asteraceae) comprises 123 species distributed across 24 genera (Carlquist *et al.*, 2003). The continental species are commonly referred to as 'tarweeds' with the Hawaiian species known as 'silverswords' alliance. The 33 Hawaiian species form a clade comprising three genera; *Argyroxiphium* DC. (5 spp.), *Dubautia* Gaudich. (26 spp.), and *Wilkesia* A.Gray (2 spp.) (Baldwin *et al.*, 1991; Baldwin & Sanderson, 1998; Baldwin & Wessa, 2000). Like *Echium*, the Hawaiian silversword radiation shows evidence of insular woodiness and disparification of insular growth forms including shrubs, treelets, lianas, acaulescent and candelabra rosettes (Baldwin, 1997) with a smaller radiation with parallel evolution of insular woodiness on the Californian islands in the genus *Deinandra* Greene (Baldwin, 2007), while the vast majority of the continental tarweeds are ephemeral low elevation herbs occupying seasonally dry mainland habitats. Although a suite of phylogenies that sample members of the Madiinae have been published (Baldwin *et al.*, 1991; Baldwin, 1997; Baldwin *et al.*, 1998; Baldwin & Sanderson, 1998; Barrier *et al.*, 1999; Baldwin & Wessa, 2000; Baldwin, 2007) none included the comprehensive sampling of both mainland and island species needed for comparative analysis of species and trait diversification rates (but see: Landis *et al.*, 2018). We generated a time-calibrated phylogenetic tree for the Silverswords–Tarweeds using the ITS marker for as many members of Madiinae with available data in GenBank (GenBank accession numbers are provided in table S1). Sequences were aligned using MUSCLE v3.6 (Edgar, 2004). Alignments were visually crosschecked and ambiguous fragments were excluded using Geneious v.6.0.5. We simultaneously estimated the topology and divergence times using BEAST v1.8 under an uncorrelated lognormal relaxed-clock model, using the general time-reversible (GTR) substitution rate model and Γ-distributed rates among sites with a proportion of invariant sites to describe the rate heterogeneity among sites. In the absence of available fossil calibrations, we used a calibration constraint of 15 Ma for the most recent common

ancestor of the silversword alliance and its Californian sister group, as justified by Baldwin and Sanderson (1998) and later used by Baldwin (2007). While this approach may not provide accurate estimates of absolute divergence times (but cf. Landis *et al.*, 2018), this will not affect our downstream rates analyses as our inferences are based on relative divergence times. We ran four independent MCMC runs of 50 × $10^6$ generations and checked for convergence between runs, and effective sample sizes of >200 for all parameters in Tracer 1.5. We combined the four runs in LogCombiner after discarding the first 10% generations (burnin) in each run before annotating the MCC tree using TreeAnnotator. The MCC tree was used in all subsequent comparative analyses after pruning all subspecies.

Our time-calibrated phylogeny of the Madiinae is concordant with previous phylogenetic hypotheses in both topology and age estimates, with a crown age estimate for the silverswords of ca. 3.6 Ma and for the tarweeds from CA silands of ca. 1.2 Ma (table 1, Fig. S4). We recovered the Hawaiian silversword taxa as monophyletic matching the phylogenies of Baldwin and Sanderson (1998) and Barrier *et al.* (1999) and also recovered the CA island clade (Fig. S4) presented by Baldwin (2007). Our finding that the two species of *Anisocarpus* Nutt. are sister to the silversword clade is concordant with the phylogeny of Baldwin and Wessa (2000) and Landis *et al.* (2018). We find that within the silversword clade *Wilkesia* is nested within *Dubautia*, which together are sister to *Argyroxiphium*, matching the previously most comprehensively sampled topology for the silverswords of Baldwin and Sanderson (1998), but differs from the groupings recently recovered by Landis *et al.* (2018), in which *Argyroxiphium* is nested within *Dubautia*.

Trait data were obtained from the Monograph of the Hawaiian Madiinae (Carr, 1985), the Jepson manual (Baldwin *et al.*, 2012), the manual of the flowering plants of Hawai'i, (Wagner *et al.*, 1999) and the monograph of tarweeds and silverswords (Carlquist *et al.*, 2003).

**Notes S1 *Hypericum* traits**: We are relaying on mean plant height as proxy for growth form evolution, which might introduce bias into comparisons of overall disparity between *Hypericum* clades because not all species descriptions contain measures of min and max plant height (Robson, 1987; 1996). For example, the North American non-island species *H. chapmanii* W.P.Adams is descried as "up to 4 m tall" in Robson (1996: p 112), whereas the Andean sky island species *H. laricifolium* Juss. is described as "(0.1–) 0.3–3 (–6) m tall" (Robson, 1987: p 47). For these two species (and *H. densiflorum* Pursh, *H. frondosum* Michx., *H irazuense* Kunze ex N.Robson, *H lycopodioides* Triana & Planch.) we evaluated additional herbarium specimens and calculated as most likely actual mean plant height 190 cm for *H. chapmanii*, and 315 cm for *H. laricifolium* that, as

adult plant, generally grows up to 3–4 (–6) m height (NM Nürk, pers. observations). Nevertheless, overall disparity of growth form might be overestimated for the non-island species and potentially underestimated for the sky island species of *Hypericum*.

References SI


**Baldwin BG 1997.** Adaptive radiation of the Hawaiian silversword alliance: congruence and conflict of phylogenetic evidence from molecular and non-molecular investigations. In: Givnish TJ, Sytsma KJ eds. *Molecular evolution and adaptive radiation*. London: Cambridge University Press, UK, 103–128.

**Baldwin BG. 2007.** Adaptive radiation of shrubby tarweeds (*Deinandra*) in the California Islands parallels diversification of the Hawaiian silversword alliance (Compositae-Madiinae). *American Journal of Botany* **94**(2): 237–248.

**Baldwin BG, Crawford DJ, Francisco-Ortega J, Kim SC, Sang T, Stuessy T 1998.** Molecular phylogenetic insights on the origin and evolution of oceanic island plants. In: Soltis D, Soltis P, Doyle JJ eds. *Molecular Systematics of Plants. II. DNA Sequencing*. Boston, USA: Kluwer Academic Publishers, 410–441.

**Baldwin BG, Goldman DH, Vorobik LA. 2012.** *The Jepson manual: vascular plants of California*. Berkeley, Calif.: University of California Press.

**Baldwin BG, Kyhos DW, Dvorak J, Carr GD. 1991.** Chloroplast DNA evidence for a North-American origin of the Hawaiian silversword alliance (Asteraceae). *Proceedings of the National Academy of Sciences of the United States of America* **88**(5): 1840–1843.

**Baldwin BG, Sanderson MJ. 1998.** Age and rate of diversification of the Hawaiian silversword alliance (Compositae). *Proceedings of the National Academy of Sciences of the United States of America* **95**(16): 9402–9406.

**Baldwin BG, Wessa BL. 2000.** Origin and relationships of the tarweed-silversword lineage (Compositae-Madiinae). *American Journal of Botany* **87**(12): 1890–1908.

**Barneby RC. 1989.** *Intermountain flora: vascular plants of the intermountain West, U.S.A. III, part B: Fabales*. New York: New York Botanical Garden.

**Barrier M, Baldwin BG, Robichaux RH, Purugganan MD. 1999.** Interspecific hybrid ancestry of a plant adaptive radiation: allopolyploidy of the Hawaiian silversword alliance (Asteraceae) inferred from floral homeotic gene duplications. *Molecular Biology and Evolution* **16**(8): 1105–1113.

**Böhle UR, Hilger HH, Martin WF. 1996.** Island colonization and evolution of the insular woody habit in *Echium* L. (Boraginaceae). *Proceedings of the National Academy of Sciences of the United States of America* **93**(21): 11740–11745.

**Bramwell D. 1972.** A revision of the genus *Echium* in Macaronesia. *Lagascalia* **2**(1): 37–115.

**Carlquist SJ, Baldwin BG, Carr GD. 2003.** *Tarweeds & silverswords: evolution of the Madiinae (Asteraceae)*. St. Louis, U.S.A.: Missouri Botanical Garden Press.

**Carr GD. 1985.** Monograph of the Hawaiian Madiinae (Asteraceae) : *Argyroxiphium*, *Dubautia*, and *Wilkesia*. *Allertonia* **4**(1): 1–123.

**Chandler MEJ. 1962.** *The lower Tertiary floras of Southern England. II, Flora of the Pipe-clay Series of Dorset (Lower Bagshot)*. London, UK: British Museum Natural History.

**Darnell MK, Thomasson JR. 2007.** First equid remains from the late Miocene *Prolithospermum johnstonii-Nassella pohlii* assemblage zone stratotype locality, Ellis County, Kansas. *Transactions of the Kansas Academy of Science* **110**(1 & 2): 10–15.

**Dorofeev PI. 1963.** *Tretichnye flory Zapadnoi Sibiri [The tertiary floras of western Siberia]*. Moskva: Izd-vo Akademii nauk SSSR.

**Drummond AJ, Suchard MA, Xie D, Rambaut A. 2012.** Bayesian Phylogenetics with BEAUti and the BEAST 1.7. *Molecular Biology and Evolution* **29**(8): 1969–1973.

**Drummond CS, Eastwood RJ, Miotto STS, Hughes CE. 2012.** Multiple continental radiations and correlates of diversification in *Lupinus* (Leguminosae): testing for key innovation with incomplete taxon sampling. *Systematic Biology* **61**(3): 443–460.

**Edgar RC. 2004.** MUSCLE: multiple sequence alignment with high accuracy and high throughput. *Nucleic Acids Research* **32**(5): 1792–1797.

**Gabel ML. 1987.** A Fossil *Lithospermum* (Boraginaceae) from the Tertiary of South-Dakota. *American Journal of Botany* **74**(11): 1690–1693.

**García-Maroto F, Manas-Fernández A, Garrido-Cárdenas JA, López Alonso D, Guil-Guerrero JL, Guzmán B, Vargas P. 2009.** Delta6-desaturase sequence evidence for explosive Pliocene radiations within the adaptive radiation of Macaronesian *Echium* (Boraginaceae). *Molecular Phylogenetics and Evolution* **52**(3): 563–574.

**Gladstones JS. 1974.** *Lupins of the Mediterranean region and Africa*. Perth: Govt. Pr.

**Hughes CE, Atchison GW. 2015.** The ubiquity of alpine plant radiations: from the Andes to the Hengduan Mountains. *New Phytologist*: DOI: 10.1111/nph.13230.

**Hughes CE, Eastwood R. 2006.** Island radiation on a continental scale: exceptional rates of plant diversification after uplift of the Andes. *Proceedings of the National Academy of Sciences of the United States of America* **103**(27): 10334–10339.

**Huzioka K, Takahasi E. 1970.** *The Eocene flora of the Ube coal-field, southwest Honshu, Japan*. Akita, Japan: Akita University, Mining College.

**Kearse M, Moir R, Wilson AC, Stones-Havas S, Cheung M, Sturrock S, Buxton S, Cooper A, Markowitu S, Duran C, et al. 2011.** Geneious Basic: an integrated and extendable desktop software platform for the organization and analysis of sequence data. *Bioinformatics* **28**(12): 1647–1649.

**Landis MJ, Freyman WA, Baldwin BG. 2018.** Retracing the Hawaiian silversword radiation despite phylogenetic, biogeographic, and paleogeographic uncertainty. *Evolution* **72**(11): 2343–2359

**Mai DH, Walther H. 1991.** *Die oligozänen und untermiozänen Floren Nordwest-Sachsens und des Bitterfelder Raumes*. Dresden, Germany: Staatliches Museum für Mineralogie und Geologie zu Dresden.

**McVaugh R, Anderson WR. 1987.** *Flora Novo-Galiciana: a descriptive account of the vascular plants of Western Mexico*. Ann Arbor: University of Michigan Press.

**Nevado B, Atchison GW, Hughes CE, Filatov DA. 2016.** Widespread adaptive evolution during repeated evolutionary radiations in New World lupins. *Nature Communications* DOI: 10.1038/ncomms12384

**Nürk NM, Blattner FR. 2010.** Cladistic analysis of morphological characters in *Hypericum* (Hypericaceae). *Taxon* **59**(5): 1495–1507.

**Nürk NM, Madriñán S, Carine MA, Chase MW, Blattner FR. 2013a.** Molecular phylogenetics and morphological evolution of St. John's wort (*Hypericum*; Hypericaceae). *Molecular Phylogenetics and Evolution* **66**(1): 1–16.

**Nürk NM, Michling F, Linder HP. 2018.** Are the radiations of temperate lineages in tropical alpine ecosystems pre-adapted? *Global Ecology and Biogeography* **27**(3): 334–345.

**Nürk NM, Scheriau C, Madriñán S. 2013b.** Explosive radiation in high Andean *Hypericum* – Rates of diversification among New World lineages. *Frontiers in Genetics* **4**(175): 1–14.

**Nürk NM, Uribe-Convers S, Gehrke B, Tank DC, Blattner FR. 2015.** Oligocene niche shift, Miocene diversification - Cold tolerance and accelerated speciation rates in the St. John's Worts (*Hypericum*, Hypericaceae). *Bmc Evolutionary Biology* **15**(80): 1–13.

**Ozaki K. 1991.** *Late Miocene and Pliocene floras in Central Honshu, Japan*. Yokohama, Japan: Kanagawa Prefectural Museum.

**Rambaut A, Drummond AJ 2007**. Tracer v1.4: Available from http://beast.bio.ed.ac.uk/Tracer

**Robson NKB. 1987.** Studies in the genus *Hypericum* L. (Guttiferae): 7. Section 29. *Brathys* (part 1). *Bulletin of the British Museum (Natural History), Botany* **16**(1): 1–106.

**Robson NKB. 1990.** Studies in the genus *Hypericum* L. (Guttiferae): 8. Sections 29. *Brathys* (part 2) and 30. *Trigynobrathys*. *Bulletin of the Natural History Museum. Botany series. London* **20**(1): 1–151.

**Robson NKB. 1996.** Studies in the genus *Hypericum* L. (Guttiferae): 6. Sections 20. *Myriandra* to 28. *Elodes*. *Bulletin of the Natural History Museum. Botany series. London* **26**(2): 75–217.

**Robson NKB. 2012.** Studies in the genus *Hypericum* L. (Hypericaceae) 9. Addenda, corrigenda, keys, lists and general discussion. *Phytotaxa* **72**: 1–111.

**Romeiras MM, Paulo OS, Duarte MC, Pina-Martins F, Cotrim MH, Carine MA, Pais MS. 2011.** Origin and diversification of the genus *Echium* (Boraginaceae) in the Cape Verde archipelago. *Taxon* **60**(5): 1375–1385.

**Stamatakis A. 2006.** RAxML-VI-HPC: maximum likelihood-based phylogenetic analyses with thousands of taxa and mixed models. *Bioinformatics* **22**(21): 2688–2690.

**Talavera S, Andrés C, Arista M, Fernández Piedra MP, Gallego MJ, Ortiz PL, Romero Zarco C, Salgueiro FJ, Silvestre S, Quintanar A. 2012.** *Flora Iberica. Vol. XI, Gentianaceae-Boraginaceae*. Madrid: Real Jardín Botánico, CSIC.

**Wagner WL, Herbst DR, Sohmer SH. 1999.** *Manual of the flowering plants of Hawai'i*. Honolulu, Hawaii, United States: University of Hawaii Press; revised edition.
